# Mental, behavioural, and developmental disorders among U.S. Children with and without heart conditions, 2016–2021

**DOI:** 10.1017/S1047951125100760

**Published:** 2025-08-20

**Authors:** Amanda N. Dorsey, Karrie F. Downing, Melissa Danielson, Vijaya Kancherla, Matthew E. Oster, Sherry L. Farr

**Affiliations:** 1 Department of Epidemiology, Rollins School of Public Health, Emory University, Atlanta, GA, USA;; 2 Centers for Disease Control and Prevention, National Center on Birth Defects and Developmental Disabilities, Atlanta, GA, USA;; 3 Children’s Healthcare of Atlanta, Atlanta, GA, USA; 4 Emory University School of Medicine, Atlanta, GA, USA

**Keywords:** Paediatric cardiology, heart conditions, neurodevelopment

## Abstract

**Background:**

Children with heart conditions, particularly CHDs, may experience adverse neurodevelopmental and psychosocial outcomes. Our study aimed to: (1) compare national prevalence of mental, behavioural, and developmental disorders among children by heart condition status and (2) identify associated characteristics among children with heart conditions.

**Methods:**

Nationally representative data from the National Survey of Children’s Health (2016–2021) on U.S. children aged 6–17 years without Down syndrome were analysed. Caregivers reported whether a healthcare provider told them their child has ever had a heart condition or currently has depression, anxiety, ADHD, behavioural, or conduct problems, Tourette syndrome, autism spectrum disorder, developmental delay, intellectual disability, learning disability, or a speech or other language disorder. Logistic regression analysis compared disorder prevalence by heart condition status and, among children with heart conditions, assessed whether disorders were associated with demographic and contextual characteristics.

**Results:**

Among 3,440 children with heart conditions, 42% had an examined disorder, compared to 23% of 133,280 children without heart conditions (adjusted prevalence ratio = 1.8; 95% confidence interval: 1.7, 2.0). Each disorder was more prevalent among children with versus without heart conditions (adjusted prevalence ratio range: 1.9 to 5.1), with anxiety (22.1%), ADHD (20.4%), and learning disabilities (19.6%) most common. Among children with heart conditions, disorders were consistently associated with an increased number of adverse childhood experiences.

**Conclusion:**

These findings support clinical guidelines recommending neurodevelopmental and mental health screening and interventions for children with heart conditions and can be used as a national baseline to gauge progress of guideline implementation.

## Introduction

Among paediatric heart conditions, CHD are the most common condition,^1^ affecting almost one in every 100 births in the United States of America.^2^ Children with CHD experience differences in neurological development, such as delayed cerebral maturation, increased brain injuries and lesions, and structural abnormalities.^3,4^ In addition, social and contextual factors may influence risk of poor psychosocial development among children and adolescents with CHD.^5,6^ For these reasons, children with CHD may be at an increased risk for mental, behavioural, or developmental disorders, including anxiety,^7–10^ depression,^7,8^ attention-deficit/hyperactivity disorder (ADHD),^8,9,11–14^ behavioural concerns,^7,9,15^ autism spectrum disorder, hereafter written as autism,^11,12,16,17^ intellectual disability,^11,12^ and developmental delay.^18^

Only two U.S. nationally representative studies have examined this issue;^12,19^ however, these studies have not comprehensively examined all mental, behavioural, and developmental disorders, and both use data from before 2012. The current study seeks to address this knowledge gap by: 1) estimating the prevalence of mental, behavioural and developmental disorders among a nationally representative sample of U.S. children with and without heart conditions, such as CHD and 2) assessing demographic and contextual characteristics associated with these disorders among U.S. children with heart conditions.

## Materials and method

We utilized data from the National Survey of Children’s Health, conducted annually from 2016–2021 by the U.S. Census Bureau on behalf of the Health Resources & Services Administration Maternal & Child Health Bureau.^20^ The National Survey of Children’s Health generates a population-based cross-sectional sample of U.S. children 0–17 years old. The survey includes weights to allow for the generation of nationally representative estimates. Further information on the National Survey of Children’s Health is available elsewhere.^21–26^

Given that many mental, behavioural, and developmental disorders do not emerge or are not recognised in infancy and early childhood,^27^ the analytic sample was restricted to children aged 6–17 years. Down syndrome is commonly comorbid with congenital heart conditions^28^ and may influence an individual’s development more strongly than the heart condition itself; thus, the analytic sample was also limited to children without a caregiver-reported Down syndrome diagnosis.

Children were categorised as having a heart condition if their caregiver responded “Yes” to the question, “Has a doctor or other health care provider EVER told you that this child has a heart condition?” To understand what proportion of children with heart conditions had congenital heart conditions, the percentage of children with heart conditions who were born with their condition was calculated using 2020 and 2021 data, the first two National Survey of Children’s Health surveys to ask this question. Children were categorised as having a mental, behavioural or developmental disorder if the caregiver reported being told by a healthcare provider (or educator, for relevant outcomes) that the child currently has a mental, emotional, or behavioural disorder (depression, anxiety problems, ADHD, behavioural or conduct problems, or Tourette syndrome) or a developmental disorder (autism, developmental delay, intellectual disability, learning disability, or speech or other language disorder). Each disorder was considered individually, in addition to composite measures of whether the child had any mental, emotional, or behavioural disorder, any developmental disorder, or any examined disorder.

The child’s sex, race and ethnicity, age, health insurance coverage, and adverse childhood experiences, family income as a percent of the federal poverty level, primary caregiver marital status, and highest level of caregiver education were all explored for potential associations with examined disorders among children with heart conditions. Race and ethnicity were included as a social construct, not as biological indicators. Similar to other analyses,^29,30^ adverse childhood experience scores were categorised as 0, 1, 2–3, or 4 or more. Children missing data on heart condition status, any examined disorder, or any demographic and contextual characteristics were excluded.

Demographic characteristics of the analytic sample were described according to heart condition status. Rao-Scott chi-square p values with an alpha of 0.05 were used to determine any statistical differences in demographic characteristics by heart condition status. The weighted prevalence of each examined disorder was also calculated by heart condition status. Accompanying 95% confidence intervals were calculated using Taylor Series. Logistic regression analysis using the predicted marginal approach was used to calculate adjusted prevalence ratios and 95% confidence intervals for examined disorders by heart condition status, controlling for the child’s sex, race and ethnicity, family income, and the highest level of caregiver education (Supplemental Figure S1). Logistic regression analysis was also used to examine associations between each demographic and contextual characteristics and each examined disorder outcome among children with a caregiver-reported heart condition. A directed acyclic graph was created for each demographic and contextual characteristic to inform confounders included in each model (Supplemental Figure S2).^31^

Two sensitivity analyses were conducted, excluding 1) children with a caregiver-reported genetic condition (aside from children with Down syndrome who were already excluded) and 2) National Survey of Children’s Health data from the years impacted by the COVID-19 pandemic (2020 and 2021). For secondary analyses, the weighted percentages of children with≥1 examined disorder who had the following, according to heart condition status, were calculated: (1) a special education or early intervention plan; (2) taken any medication because of difficulties with their emotions, concentration, or behaviour; and (3) received any treatment or counselling from a mental health professional.

Weights and design parameters were included throughout analyses to account for complex sampling and to produce nationally representative population-based estimates. Human subjects review was not required because this was a secondary analysis of publicly available, de-identified datasets.

## Results

An unweighted total of 154,845 children aged 6–17 years without caregiver-reported Down syndrome was identified in the 2016–2021 National Survey of Children’s Health. After excluding children with missing data, an analytic sample of 136,720 children remained (Supplementary Figure S3). Some characteristics differed between those included and excluded from the analysis. Notably, among children with and without heart conditions, those included were less likely than those excluded to have ≥1 examined disorder (Supplemental Table S1). Within the analytic sample, 3,440 children had a caregiver-reported heart condition, representing a national weighted estimate of 972,486 children with heart conditions in the United States of America or 2.3% of all U.S. children (Table 1). Only two National Survey of Children’s Health survey iterations (year 2020 and year 2021) during this study’s timeframe asked, “was this child born with the [heart] condition”; 88.7% of these children were reported to have been born with their heart condition. When the caregiver reported that the child *currently* has the heart condition, 77.0% classified it as mild, 15.3% as moderate, and 7.8% as severe. Compared to children without heart conditions, those with heart conditions were more likely to have experienced a higher number of adverse childhood experiences; race and ethnicity and health insurance coverage were differentially distributed (corresponding *p* values <0.05; Table 1).

**Figure 1.**
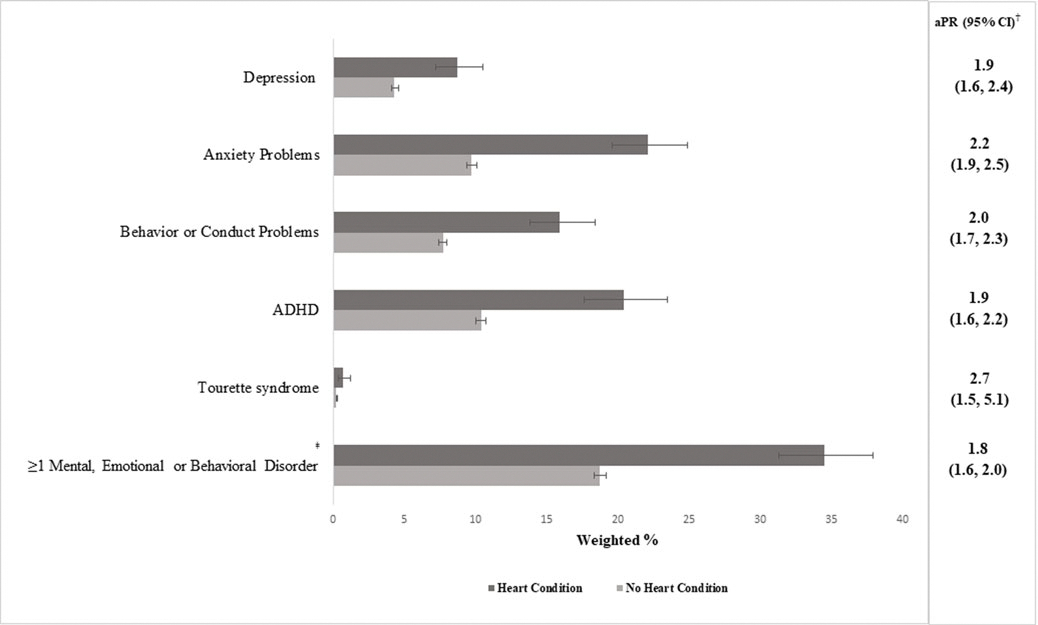
Prevalence of current mental, behavioral, and developmental disorders among children 6–17 years old with and without heart conditions, National Survey of Children’s Health, United States, 2016–2021. aPR = adjusted prevalence ratio; CI = confidence interval. ^†^Prevalence ratio adjusted for the child’s sex, race and ethnicity, family income, and highest level of caregiver education. ^‡^Any current depression, anxiety problems, ADHD, behavioral or conduct problems, and/or tourette syndrome.

Experiencing ≥1 examined disorder was 1.8 times (95% confidence interval: 1.7–2.0) as prevalent among children with heart conditions (42.0%) compared to those without (23.1%). The prevalence of ≥1 mental, emotional, or behavioural disorder was 34.5% among children with heart conditions and 18.7% among those without (adjusted prevalence ratio: 1.8, 95% confidence interval: 1.6–2.0) (Figure 1). Each specific mental, emotional, or behavioural disorder was significantly more prevalent among children with heart conditions compared to those without, with adjusted prevalence ratios ranging from 1.9 for depression (confidence interval: 1.6–2.4) and ADHD (confidence interval: 1.6–2.2) to 2.7 for Tourette syndrome (confidence interval: 1.5–5.1). The most common mental, emotional, or behavioural disorder among children with heart conditions was anxiety problems (22.1%), then ADHD (20.4%) (Figure 1).

**Figure 2.**
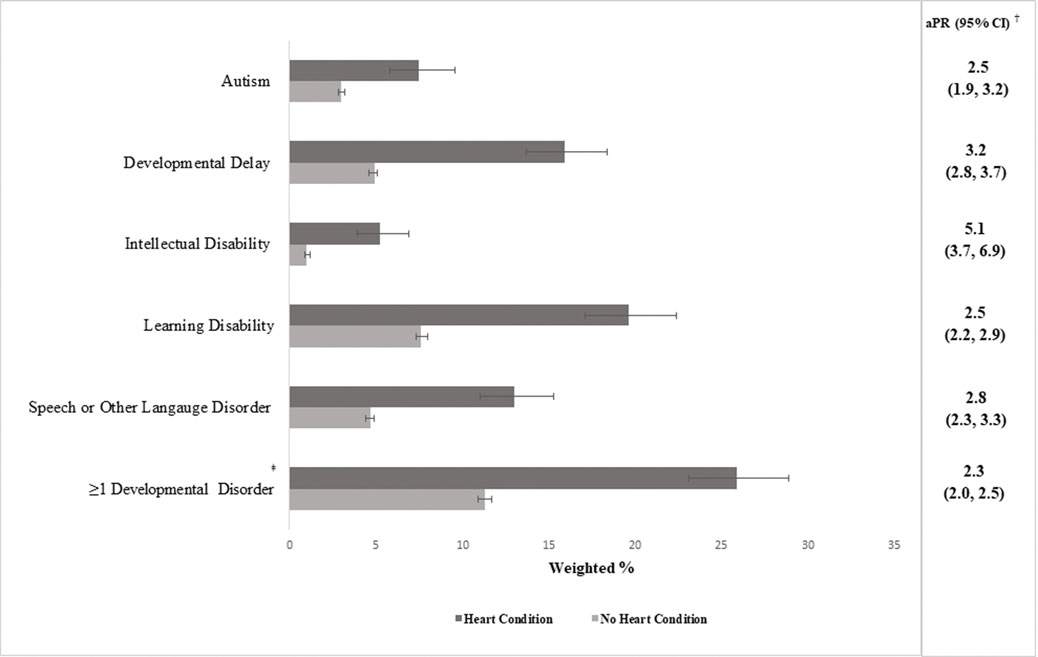
Prevalence of current developmental disorders among children 6–17 years old with and without heart conditions, National Survey of Children’s Health, United States, 2016–2021. aPR = adjusted prevalence ratio; autism = autism spectrum disorder; CI = confidence interval. ^†^Prevalence ratio adjusted for the child’s sex, race and ethnicity, income, and the highest level of caregiver education. ^‡^Any current autism, developmental delay, intellectual disability, learning disability, and/or speech or other language disorder.

Currently experiencing ≥1 developmental disorder was 2.3 times (95% confidence interval: 2.0–2.5) as prevalent in children with heart conditions (25.9%) compared to those without (11.3%; Figure 2). All specific developmental disorders were significantly more prevalent among children with, versus without, heart conditions, with adjusted prevalence ratios ranging from 2.5 for autism (confidence interval: 1.9–3.2) and learning disability (confidence interval: 2.2–2.9) to 5.1 for intellectual disability (confidence interval: 3.7–6.9). The most common developmental disorders among children with heart conditions were learning disability (19.6%) and developmental delay (15.9%) (Figure 2).

Among children with heart conditions, male sex, older age (12–17 years), having divorced, separated, or widowed caregivers, having caregivers with less than a college education, and higher adverse childhood experience scores were significantly associated with having ≥1 mental, emotional, or behavioural disorder (adjusted prevalence ratio range: 1.3–3.4; Table 2a). The largest adjusted prevalence ratios were observed with adverse childhood experience scores; 67.3% of children with 4 or more adjusted prevalence ratios had ≥1 mental, emotional, or behavioural disorder compared to 20.0% of children with no adverse childhood experiences (adjusted prevalence ratio = 3.4; 95% confidence interval: 2.6–4.4). Factors associated with having a current mental, emotional, or behavioural disorder varied according to the type of disorder. Males were 1.7-times as likely as females to have ADHD (95% confidence interval: 1.2–2.4) and 1.8-times as likely as females to have behavioural or conduct problems (95% confidence interval: 1.3–2.5). ADHD was 1.7-times (95% confidence interval: 1.2–2.4) as prevalent among Non-Hispanic Black children than Non-Hispanic white children, but no other significant associations were found for race and ethnicity. All mental, emotional, and behavioural disorders were more prevalent among children 12–17 years old compared to those 6–11 years old, except for behavioural or conduct problems. Children whose family income was less than 200% of the federal poverty level were significantly more likely to have depression (adjusted prevalence ratio range: 2.3–2.5). Children with caregivers who were divorced, separated, or widowed, compared to married or cohabiting, had 1.4–1.9-times the prevalence of all mental, emotional, and behavioural disorders, except for anxiety problems. Having caregivers with some college or an associate degree, versus a college degree or higher, was significantly associated with all mental, emotional, and behavioural disorders except ADHD (adjusted prevalence ratio range: 1.3–1.8). Public, compared to private, insurance coverage was associated with behavioural or conduct problems (adjusted prevalence ratio = 1.7; 95% confidence interval: 1.0–2.8). Finally, a positive dose–response relationship was observed between adverse childhood experience score and all types of mental, emotional, and behavioural disorders examined (adjusted prevalence ratio range: 1.6–6.5), where adjusted prevalence ratios for each disorder increased with an increasing number of adverse childhood experiences.

When examining developmental disorders, male sex, Non-Hispanic Black race, lower caregiver education, and higher adverse childhood experience scores were significantly associated with having ≥1 developmental disorder among children with heart conditions (adjusted prevalence ratio range: 1.2–2.2; Table 2b). The largest adjusted prevalence ratios were observed with adverse childhood experience scores; 44.4% of children with 4 or more adverse childhood experiences had ≥1 developmental disorder compared to 18.2% of children with no adverse childhood experiences (adjusted prevalence ratio = 2.2; 95% confidence interval: 1.5–3.0). Demographic and contextual characteristics associated with current developmental disorders also varied by type of disorder. All specific developmental disorders examined were 1.4–2.9-times as prevalent in males compared to females, although 95% confidence intervals were wide and included 1.0 for intellectual disability. Intellectual disability, learning disability, and speech or other language disorder were 2.4 (95% confidence interval: 1.2–5.0), 1.6 (95% confidence interval: 1.1–2.3), and 1.6 (95% CI: 1.0–2.5) times, respectively, as prevalent in Non-Hispanic Black children than Non-Hispanic white children with heart conditions, though the 95% confidence interval included 1.0 for speech or other language disorder. Children 12–17 years old were 1.8-times as likely than those 6–11 years old to have autism (95% confidence interval: 1.1–3.0) and 1.4 times as likely to have a learning disability (95% confidence interval: 1.1–1.8), but 0.7-times as likely to have a speech or other language disorder (95% confidence interval: 0.5–1.0). Family income and primary caregiver marital status were not significantly associated with developmental disorders. Children of caregivers with a high school education or less or some college or an associate degree, compared to a college degree or higher, were significantly or marginally significantly as likely to have each developmental disorder except intellectual disability (adjusted prevalence ratio range: 1.4–2.1). Public, compared to private, health insurance coverage was significantly and positively associated with developmental delay (adjusted prevalence ratio: 1.6; 95% confidence interval: 1.1–2.5). Lastly, a dose–response relationship was observed with adverse childhood experience scores and autism, developmental delay, and learning disability (adjusted prevalence ratio range: 1.2–4.9), where children with increasing adverse childhood experience scores had a higher prevalence of these developmental disorders.

Primary results did not substantially change when children with any reported genetic conditions were excluded from the analysis, nor when excluding data from years 2020 and 2021 when the COVID-19 pandemic may have impacted associations (Supplemental Table S2–S5). Results assessing associations with demographic and contextual characteristics without data from years 2020 and 2021 are not shown due to a substantially reduced sample size.

Among children experiencing ≥1 examined disorder, 59.2% of those with heart conditions and 47.5% of those without reported receiving early intervention or special education in their lifetime (*p* < 0.001). About 36.8% of children with and 37.4% of children without heart conditions took medication in the past 12 months because of difficulties with their emotions, concentration, or behaviour (*p* = 0.80). Receipt of any treatment or counselling from a mental health professional in the past 12 months did not differ by heart condition status (*p* = 0.66). About 40% received any treatment or counselling from a mental health professional in the past 12 months, and 7% needed but did not receive it.

## Discussion

In nationally representative U.S. data from the 2016–2021 National Survey of Children’s Health, over two in five children with heart conditions had one or more examined disorder, twice as high as children without heart conditions. Each specific disorder examined was approximately two to five times more prevalent among children with, versus without, heart conditions, with the greatest adjusted prevalence ratios for intellectual disability, developmental delay, and speech or other language disorder. Among children with heart conditions, over one in three had any mental, emotional, or behavioural disorder, and over one in four had any developmental disorder. Anxiety problems, ADHD, and learning disability were the most common, each affecting approximately one in five children with heart conditions. Demographic and contextual characteristics were associated with each individually examined disorder among children with heart conditions, and included male sex, Non-Hispanic Black race, older age, public health insurance, lower family income, divorced, separated, or widowed primary caregiver marital status, lower caregiver education, and adverse childhood experience score. Adverse childhood experience score was consistently and most strongly associated with these disorders, exhibiting a positive dose–response effect for almost all disorders. Sensitivity analyses did not notably change the results. Among those with ≥1 disorder, some indicators of resource use did not differ according to heart condition status, but early intervention or special education services were more common in children with heart conditions.

These National Survey of Children’s Health results examining children with heart conditions, including CHD, agree with previous studies suggesting that children with CHD are at increased risk for mental, behavioural, and developmental disorders.^7–9,11,12,18^ However, our analysis is one of the first to examine Tourette syndrome in this population, finding that 0.7% of children with heart conditions also have Tourette syndrome, almost three times as prevalent compared to children without heart conditions. We are also one of the first to provide a U.S. prevalence of speech or other language disorders among children with heart conditions, though previous studies have identified elevated risks.^32,33^

Additionally, this study identified associations with specific disorders, including lower family income with anxiety,^10^ older age with autism,^12^ and Non-Hispanic Black race and ethnicity with intellectual disability,^11^ not examined in previous studies.^7,8,14^ Differences in findings between previous studies and this one may be due to different sampling methods and disorder classification, as this study uses caregiver-reported data. Findings from other studies requiring formal diagnoses might be affected by inequitable access to diagnostic services.^8,34^ Gonzalez et al. (2021) found that children who were not reported as white with CHD were less likely than their Non-Hispanic white counterparts to have a diagnosis code or documented treatment for anxiety and depression.^8^ Gonzalez and colleagues argued that differences in disorder diagnoses by demographic and contextual characteristics reflected systemic disparities in access to mental, behavioural, or developmental disorder care and diagnoses, rather than physiologic differences.^8^ Additionally, while adverse child-hood experiences scores are associated with poor mental and developmental outcomes in general populations of children,^35^ our study is one of the first to observe this association and find an increased prevalence of adverse childhood experiences among children with heart conditions.

Our results support guidelines from organisations like the American Heart Association^36,37^ recommending neurodevelopmental and mental health interventions for children with CHD and their caregivers during prenatal care,^38^ infancy,^36^ and across the lifespan.^37^ To benefit the mental health of individuals with CHD and their families, some recommend integrating psychologists into CHD care.^37^ Healthcare professionals including paediatric cardiologists have explored screening tools to measure mental well-being among their patients with CHD^39^ and developed interventions to support affected individuals.^40^ Our findings can be used as a baseline to gauge progress as these guidelines are implemented.

Our study has several limitations. This analysis examined children with congenital and acquired heart conditions, not limited to CHD, leading to some heterogeneity among the children with heart conditions. However, 2020 and 2021 National Survey of Children’s Health data showed that almost 9 in 10 children with heart conditions were born with the condition. Heart condition, examined disorders, and all demographic and contextual characteristics are caregiver-reported and are subject to misclassification. The child’s cardiac surgical history was not available.^4,8,9^ We could not discern temporality between demographic and contextual characteristics and disorder outcomes. Due to missing data, 11.7% of children in National Survey of Children’s Health were excluded. Disorder prevalence was slightly higher among excluded compared to included children, so we may have underestimated the prevalence of examined disorders. Detection bias may have also affected disorder prevalence; 91.0% of children with heart conditions in our analytic sample saw a health care professional in the past 12 months, compared to 80.1% of children without heart conditions; therefore, children with heart conditions may have had more opportunities to have a disorder diagnosed. However, this analysis provides nationally representative findings on prevalence and factors associated with these disorders among children with heart conditions in the United States of America. Our sample of 3,440 children with heart conditions is a large number with more recent data compared to prior studies.

Two in five U.S. children aged 6–17 years with heart conditions had one or more examined mental, behavioural, or developmental disorders, almost twice that of their peers without heart conditions. These results support national guidelines for screening children with CHD for stressors and reduced health-related quality of life and connecting impacted families with resources.^36,37,39^ Holistic support for a safe and secure environment may support the mental health and developmental well-being of children with heart conditions and their families, as adverse childhood experience score was the factor most strongly associated with examined disorders, in addition to social determinants of health such as non-Hispanic Black race and ethnicity, public health insurance coverage, and lower family income. These findings draw attention to racial and socio-economic disparities in the well-being and development of children with heart conditions in the United States of America. Screening, treating, referring, and providing ample support for families of children with heart conditions in evidence-based and cost-effective ways may help these children receive needed services and be set on a path for positive neurodevelopment and mental health.

## Supplementary Material

SUP - Dorsey - mental-behavioural-and-developmental-disorders-among-us-children-with-and-without-heart-conditions-2016-2021

**Supplementary material.** The supplementary material for this article can be found at https://doi.org/10.1017/S1047951125100760.

## Figures and Tables

**Table 1.** Characteristics of children 6–17 years old by heart condition status, National Survey of Children’s Health, United States, 2016–2021

Characteristic		Heart Condition				Chi-square p-value
		Yes		No		
		Unweighted N	Weighted Percent, (95% CI^†^)	Unweighted N	Weighted Percent, (95% CI^†^)	
Total	Total	3,440	2.3 (2.1, 2.5)	133,280	97.7 (97.5, 97.9)	
Sex	Male	1,891	51.2 (47.5, 54.9)	68,788	51.2 (50.6, 51.8)	0.99
	Female	1,549	48.8 (45.1, 52.5)	64,492	48.8 (48.2, 49.4)	
Race/ethnicity	NH White	2,480	58.1 (54.3, 61.8)	92,166	51.7 (51.1, 52.3)	0.001
	NH Black	215	12.5 (10.3, 15.0)	8,180	12.9 (12.5, 13.4)	
	Hispanic	370	21.3 (17.8, 25.4)	15,949	25.0 (24.3, 25.6)	
	Other	375	8.1 (6.6, 10.0)	16,985	10.4 (10.1, 10.7)	
Age (years)	6–11 years	1,445	49.7 (46.1, 53.4)	57,998	50.0 (49.4, 50.6)	0.90
	12–17 years	1,995	50.3 (46.6, 53.9)	75,282	50.0 (49.4, 50.6)	
Family Income (% FPL)	<100%	434	19.8 (15.9, 24.4)	14,439	18.0 (17.5, 18.6)	0.16
	100–199%	592	20.3 (16.5, 24.6)	21,000	21.4 (20.8, 21.9)	
	200–399%	1,103	31.0 (27.5, 34.6)	40,924	28.5 (28.0, 29.1)	
	>400%	1,312	28.9 (25.9, 32.1)	56,918	32.1 (31.5, 32.6)	
Primary Caregiver Marital Status	Married or living with a partner	2,715	73.4 (69.4, 77.0)	107,602	77.8 (77.2, 78.3)	0.09
	Never married	193	8.3 (6.4, 10.9)	6,374	7.2 (6.8, 7.5)	
	Divorced, separated, or widowed	532	18.3 (15.0, 22.1)	19,304	15.0 (14.6, 15.5)	
Highest Level of Caregiver Education	High school or less than high school	509	24.9 (21.3, 29.0)	20,476	28.7 (28.0, 29.4)	0.12
	Some college or associate degree	879	23.8 (21.2, 26.6)	31,057	21.8 (21.3, 22.3)	
	College degree or higher	2,052	51.3 (47.6, 54.9)	81,747	49.5 (48.9, 50.2)	
Health Insurance Coverage	Any private insurance	2,495	60.6 (56.7, 64.4)	101,838	64.5 (63.8, 65.1)	0.02
	Public insurance only	825	34.3 (30.6, 38.3)	25,584	28.8 (28.1, 29.4)	
	None	120	5.1 (3.4, 7.6)	5,858	6.8 (6.4, 7.2)	
ACE Score	0	1,604	40.6 (37.2,44.1)	74,752	53.2 (52.6, 53.8)	<0.0001
	1	818	25.3 (22.1, 28.8)	29,982	23.9 (23.4, 24.5)	
	2–3	688	23.1 (19.8, 26.6)	20,079	16.0 (15.6, 16.5)	
	4 or more	330	11.0 (9.0, 13.5)	8,467	6.9 (6.6, 7.2)	
Survey Year	2016	718	17.5 (14.8, 20.5)	30,324	16.3 (15.9, 16.7)	0.62
	2017	334	16.9 (13.8, 20.6)	13,382	16.8 (16.3, 17.4)	
	2018	489	15.6 (13.4, 18.0)	19,036	16.8 (16.4, 17.3)	
	2019	491	15.5 (13.4, 18.0)	18,412	17.0 (16.5, 17.5)	
	2020	699	16.7 (14.2, 19.6)	26,537	16.6 (16.2, 17.0)	
	2021	709	17.8 (15.2, 20.7)	25,589	16.4 (16.0, 16.8)	

ACE = adverse childhood experiences; CI = confidence interval; FPL = Federal Poverty Line; NH = Non-Hispanic. Exclusion criteria = Missing information on heart condition status, mental, behavioral, and developmentaldisorderstatus, ordemographicand contextual information.

† Confidence interval calculated using Taylor Series.

**Table 2a.** Demographic and contextual characteristics associated with mental, behavioral, and developmental disorders among children 6–17 years old with heart conditions, National survey of Children’s Health, United States, 2016–2021

Characteristic		Depression		Anxiety Problems		ADHD		Behavioral or Conduct Problems		≥ 1 Mental, Emotional, or Behavioral Disorder^†^	
		Weighted Percent (95% CI^‡^)	aPR (95% CI)	Weighted Percent (95% CI^‡^)	aPR (95% CI)	Weighted Percent (95% CI^‡^)	aPR (95% CI)	Weighted Percent (95% CI^‡^)	aPR (95% CI)	Weighted Percent (95% CI^‡^)	aPR (95% CI)
Sex^§^	Male	9.7 (7.5, 12.6)	1.28 (0.88, 1.87)	23.6 (20.2, 27.5)	1.15 (0.91, 1.46)	25.7 (22.2, 29.5)	1.73 (1.22, 2.44)	20.5 (17.4, 23.9)	1.83 (1.33, 2.51)	38.9 (34.9, 43.0)	1.30 (1.06, 1.59)
	Female	7.6 (5.8, 9.9)	ref	20.6 (17.1, 24.6)	ref	14.8 (10.7, 20.2)	ref	11.2 (8.5, 14.7)	ref	29.9 (25.0, 35.4)	ref
Race and ethnicity^§^	NH White	8.9 (7.1, 11.2)	ref	23.2 (20.2, 26.4)	ref	19.5 (16.8, 22.6)	ref	15.8 (13.4, 18.4)	ref	35.2 (31.7, 38.9)	ref
	NH Black	7.4 (4.0, 13.2)	0.83 (0.44, 1.57)	27.1 (18.7, 37.5)	1.17 (0.80, 1.70)	33.1 (23.7, 44.0)	1.69 (1.20, 2.39)	23.7 (15.4, 34.6)	1.50 (0.97, 2.32)	44.3 (34.4, 54.7)	1.26 (0.98, 1.62)
	Hispanic	6.9 (3.8, 12.3)	0.78 (0.41, 1.46)	16.6 (11.2, 23.9)	0.72 (0.48, 1.07)	15.9 (8.8, 27.1)	0.82 (0.46, 1.47)	11.2 (7.1, 17.3)	0.71 (0.44, 1.14)	28.2 (19.6, 38.7)	0.80 (0.56, 1.14)
	Other	13.4 (8.4, 20.5)	1.50 (0.91, 2.47)	21.8 (15.4, 29.8)	0.94 (0.66, 1.34)	18.8 (12.9, 26.7)	0.97 (0.65, 1.43)	17.8 (11.9, 25.7)	1.13 (0.74, 1.71)	31.2 (23.4, 40.2)	0.88 (0.66, 1.18)
Age (years) ^§^	6–11 years	3.4 (2.2, 5.2)	ref	17.1 (13.8, 21.0)	ref	16.4 (13.2, 20.1)	ref	16.1 (13.1, 19.7)	ref	27.6 (23.4, 32.1)	ref
	12–17 years	13.9 (11.3, 17.1)	4.15 (2.55, 6.74)	27.1 (23.5, 31.0)	1.58 (1.23, 2.03)	24.4 (20.0, 29.3)	1.49 (1.12, 1.98)	15.8 (12.8, 19.3)	0.98 (0.73, 1.31)	41.4 (36.8, 46.2)	1.50 (1.24, 1.82)
Family Income (FPL) ^¶^	<100%	12.4 (7.8, 19.0)	2.32 (1.29, 4.16)	23.1 (16.5, 31.4)	1.13 (0.69, 1.85)	24.7 (17.1, 34.1)	1.36 (0.84, 2.19)	19.5 (13.3, 27.7)	1.29 (0.76, 2.18)	35.7 (26.4, 46.1)	1.12 (0.77, 1.61)
	100–199%	13.0 (8.7, 19.1)	2.45 (1.38, 4.35)	25.7 (19.9, 32.6)	1.31 (0.88, 1.95)	20.1 (15.1, 26.4)	1.18 (0.78, 1.78)	21.8 (16.4, 28.4)	1.57 (0.99, 2.48)	37.8 (30.7, 45.4)	1.22 (0.93, 1.61)
	200–399%	6.5 (4.5, 9.2)	1.17 (0.73, 1.87)	22.2 (17.5, 27.7)	1.15 (0.82, 1.61)	21.2 (15.3, 28.7)	1.24 (0.81, 1.88)	13.9 (10.3, 18.4)	1.08 (0.73, 1.59)	35.7 (29.1, 42.9)	1.16 (0.88, 1.53)
	≥400%	5.6 (4.1, 7.6)	ref	18.9 (15.3, 23.2)	ref	16.8 (13.4, 20.9)	ref	11.6 (8.9, 15.2)	ref	30.3 (25.8, 35.1)	ref
Primary Caregiver Marital Status^¶^	Married or living with a partner	7.3 (5.7, 9.2)	ref	21.0 (18.3, 24.1)	ref	17.3 (14.8, 20.1)	ref	13.8 (11.7, 16.3)	ref	30.7 (27.6, 34.0)	ref
	Never married	8.8 (5.0, 15.1)	1.08 (0.53, 2.19)	22.4 (13.5, 34.8)	0.95 (0.56, 1.61)	25.4 (14.8, 40.1)	1.26 (0.75, 2.11)	22.4 (13.0, 35.9)	1.27 (0.74, 2.15)	41.8 (29.0, 55.9)	1.29 (0.91, 1.81)
	Divorced, separated, or widowed	14.3 (9.5, 21.0)	1.88 (1.22, 2.90)	26.4 (19.5, 34.6)	1.20 (0.88, 1.64)	30.5 (21.1, 41.8)	1.71 (1.14, 2.57)	21.4 (15.3, 29.1)	1.41 (0.99, 2.01)	46.4 (35.6, 57.6)	1.49 (1.13, 1.96)
Highest Level of Caregiver Education^#^	High school or less than high school	10.2 (6.4, 15.7)	1.58 (0.93, 2.68)	23.6 (17.7, 30.7)	1.23 (0.88, 1.71)	20.3 (14.7, 27.4)	1.06 (0.71, 1.60)	19.4 (13.9, 26.5)	1.68 (1.15, 2.45)	34.2 (26.8, 42.6)	1.08 (0.82, 1.43)
	Some college or associate degree	11.4 (8.4, 15.2)	1.78 (1.18, 2.67)	25.9 (21.3, 31.1)	1.32 (1.02, 1.72)	23.7 (19.2, 28.9)	1.21 (0.86, 1.69)	21.3 (17.0, 26.2)	1.79 (1.31, 2.45)	40.5 (34.9, 46.3)	1.26 (1.02, 1.55)
	College degree or higher	6.7 (5.2, 8.6)	ref	19.7 (16.5, 23.3)	ref	18.9 (14.9, 23.6)	ref	11.8 (9.5, 14.5)	ref	31.9 (27.6, 36.6)	ref
Health Insurance Coverage^||^	Any private insurance	6.9 (5.5, 8.8)	Ref	20.4 (17.6, 23.5)	ref	18.4 (14.8, 22.6)	ref	12.2 (9.9, 15.0)	ref	32.2 (28.4, 36.3)	ref
	Public insurance only	12.2 (8.9, 16.5)	1.17 (0.74, 1.85)	25.9 (20.8, 31.7)	1.21 (0.84, 1.73)	24.7 (19.8, 30.3)	1.28 (0.84, 1.97)	23.3 (18.7, 28.8)	1.71 (1.04, 2.81)	40.0 (33.5, 46.9)	1.23 (0.94, 1.62)
	None	5.7 (2.7, 11.5)	0.59 (0.25, 1.38)	17.6 (9.3, 30.7)	0.83 (0.42, 1.62)	15.5 (7.5, 29.1)	0.80 (0.37, 1.72)	10.1 (4.6, 21.0)	0.76 (0.31, 1.86)	25.0 (14.0, 40.5)	0.77 (0.44, 1.36)
ACE Score^††^	0	3.0 (2.1, 4.3)	ref	12.1 (9.8, 14.8)	ref	9.6 (7.6, 11.9)	ref	7.9 (6.1, 10.3)	Ref	20.0 (17.0, 23.4)	ref
	1	7.0 (5.0, 9.8)	2.32 (1.36, 3.96)	22.2 (17.2, 28.3)	2.03 (1.45, 2.85)	22.0 (15.3, 30.6)	2.31 (1.61, 3.32)	12.6 (9.3, 16.9)	1.55 (1.05, 2.29)	36.4 (29.1, 44.3)	1.86 (1.45, 2.38)
	2–3	13.2 (9.0, 18.9)	4.01 (2.29, 7.02)	29.6 (23.3, 36.8)	2.75 (1.98, 3.82)	25.7 (20.0, 32.4)	2.61 (1.78, 3.83)	21.0 (15.9, 27.2)	2.35 (1.51, 3.66)	42.4 (34.7, 50.5)	2.13 (1.64, 2.76)
	4 or more	23.8 (16.6, 32.9)	6.53 (3.77, 11.31)	43.4 (33.4, 53.9)	4.08 (2.88, 5.78)	45.3 (34.9, 56.2)	4.72 (3.26, 6.82)	42.4 (32.3, 53.2)	4.92 (3.27, 7.42)	67.3 (56.2, 76.7)	3.39 (2.63, 4.36)

ACE = adverse childhood experiences; aPR = adjusted prevalence ratio; CI = Confidence Interval; FPL = Federal Poverty Level; NH = non-Hispanic.

† Any current depression, anxiety problems, ADHD, behavioral orconduct problem, and/orTourette syndrome.

‡ Confidence interval calculated using Taylor Series.

§ No confounders identified for adjustment

¶ Adjusted for race and ethnicity and highest level of caregiver education.

# Adjusted for race and ethnicity.

|| Adjusted for family income.

†† Adjusted for race and ethnicity, age, family income, primary caregiver marital status, highest level of caregiver education, health insurance.

**Table 2b.** Demographic and contextual characteristics associated with developmental disorders among children 6–17 years old with heart conditions, National Survey of Children’s Health, United States, 2016–2021

Characteristic		Autism		Developmental Delay		Intellectual Disability		Learning Disability		Speech or Other Language Disorder		≥1 Developmental Disorder^†^	
		Weighted Percent (95% CI^‡^)	aPR (95% CI)	Weighted Percent (95% CI^‡^)	aPR (95% CI)	Weighted Percent (95% CI^‡^)	aPR (95% CI)	Weighted Percent (95% CI^‡^)	aPR (95% CI)	Weighted Percent (95% CI^‡^)	aPR (95% CI)	Weighted Percent (95% CI^‡^)	aPR (95% CI)
Sex^§^	Male	10.9 (8.4, 14.2)	2.85 (1.48, 5.50)	19.9 (16.7,23.6)	1.72 (1.25, 2.35)	6.4 (4.5,9.2)	1.69 (0.95, 3.00)	22.7 (19.3, 26.6)	1.38 (1.05, 1.83)	16.6 (13.6, 20.2)	1.80 (1.29, 2.51)	31.5 (27.7, 35.6)	1.57 (1.24, 1.98)
	Female	3.8 (2.1, 6.9)	ref	11.6 (8.9,15.0)	ref	3.8 (2.4,5.9)	ref	16.4 (13.0, 20.5)	ref	9.3 (7.0, 12.1)	ref	20.1 (16.4, 24.4)	ref
Race/ethnicity^§^	NH White	6.6 (4.9, 8.9)	ref	15.4 (12.7,18.4)	ref	3.8 (2.7,5.3)	ref	17.8 (15.0, 21.0)	ref	11.8 (9.7, 14.4)	ref	24.6 (21.5, 28.1)	ref
	NH Black	11.5 (5.5, 22.3)	1.73 (0.81, 3.71)	20.1 (13.4, 28.8)	1.30 (0.85, 2.00)	9.2 (4.7,17.1)	2.41 (1.16, 5.01)	27.8 (19.4, 38.1)	1.56 (1.07, 2.28)	18.8 (12.2, 28.0)	1.59 (1.00, 2.53)	36.8 (27.5,47.2)	1.49 (1.10, 2.02)
	Hispanic	8.1 (4.4, 14.6)	1.23 (0.62, 2.41)	14.1 (9.2, 21.0)	0.92 (0.58, 1.45)	6.5 (3.2,12.9)	1.71 (0.78, 3.74)	19.1 (13.2, 26.9)	1.07 (0.72, 1.59)	12.6 (8.0, 19.2)	1.06 (0.66, 1.72)	22.8 (16.3, 30.8)	0.92 (0.65, 1.31)
	Other	5.7 (2.7, 11.6)	0.86 (0.39, 1.90)	17.6 (11.7, 25.6)	1.14 (0.74, 1.77)	5.1 (2.6,9.9)	1.34 (0.63, 2.86)	21.5 (14.8, 30.0)	1.20 (0.81, 1.78)	13.7 (8.5, 21.3)	1.16 (0.70, 1.91)	26.5 (19.1, 35.5)	1.08 (0.77, 1.51)
Age (years) ^§^	6–11 years	5.3 (3.7, 7.4)	ref	16.7 (13.6, 20.3)	ref	3.9 (2.5, 6.0)	ref	16.4 (13.4, 20.0)	ref	15.3 (12.3, 18.9)	ref	25.1 (21.3, 29.4)	ref
	12–17 years	9.7 (6.9, 13.4)	1.84 (1.13, 2.97)	15.0 (12.0, 18.7)	0.90 (0.67, 1.21)	6.4 (4.4, 9.2)	1.65 (0.93, 2.94)	22.8 (19.0, 27.1)	1.39 (1.07, 1.82)	10.7 (8.3, 13.8)	0.70 (0.50, 0.98)	26.7 (22.8, 31.0)	1.06 (0.85, 1.33)
Family Income (% FPL) ^¶^	<100%	11.1 (6.1, 19.2)	1.56 (0.67, 3.60)	21.3 (14.6, 29.9)	1.26 (0.73, 2.18)	6.1 (3.3, 10.9)	1.57 (0.57, 4.34)	26.6 (18.9, 36.0)	1.21 (0.75, 1.95)	10.8 (7.0, 16.2)	0.66 (0.35, 1.28)	30.7 (22.3, 40.7)	1.04 (0.68, 1.58)
	100–199%	10.0 (6.3, 15.6)	1.47 (0.66, 3.31)	21.9 (16.3, 28.8)	1.37 (0.82, 2.28)	8.3 (4.7, 14.4)	2.22 (0.83, 5.94)	26.5 (20.4, 33.6)	1.29 (0.84, 1.99)	18.6 (13.4, 25.3)	1.25 (0.69, 2.28)	34.4 (27.4, 42.1)	1.25 (0.87, 1.80)
	200–399%	5.9 (3.3, 10.2)	1.03 (0.48, 2.22)	11.7 (8.5, 15.9)	0.83 (0.54, 1.28)	3.8 (1.9,7.5)	1.00 (0.43, 2.37)	15.6 (11.7, 20.6)	0.93 (0.62, 1.38)	12.5 (9.0, 17.1)	0.96 (0.60, 1.54)	22.0 (17.6, 27.3)	0.93 (0.68, 1.26)
	≥400%	5.0 (3.2, 7.7)	ref	12.5 (9.4, 16.3)	ref	3.8 (2.2, 6.3)	ref	14.4 (11.2, 18.4)	ref	11.2 (8.1, 15.3)	ref	20.9 (16.9, 25.6)	ref
Primary Caregiver Marital Status^¶^	Married or living with a partner	6.4 (4.8, 8.5)	ref	15.3 (13.0, 18.0)	ref	5.1 (3.6, 7.2)	ref	17.5 (14.9, 20.3)	ref	13.8 (11.4, 16.5)	ref	24.4 (21.5, 27.5)	ref
	Never married	11.1 (4.2, 26.6)	1.32 (0.55, 3.17)	18.9 (10.6, 31.6)	0.94 (0.49, 1.82)	3.5 (1.3, 9.1)	0.48 (0.14, 1.60)	29.8 (18.4, 44.5)	1.28 (0.77, 2.11)	11.3 (5.8, 20.7)	0.60 (0.29, 1.22)	34.7 (22.5, 49.3)	1.10 (0.71, 1.70)
	Divorced, separated, or widowed	10.2 (5.9, 17.1)	1.41 (0.79, 2.52)	16.6 (11.1, 24.1)	0.96 (0.64, 1.44)	6.2 (3.5, 10.7)	1.10 (0.59, 2.03)	23.7 (17.0, 32.1)	1.20 (0.84, 1.71)	10.8 (7.1, 16.2)	0.71 (0.46, 1.11)	28.0 (20.5, 36.8)	1.03 (0.75, 1.41)
Highest Caregiver Education^#^	High school or less than high school	11.0 (6.6, 18.0)	2.06 (1.07, 3.98)	21.4 (15.6, 28.6)	1.90 (1.27, 2.86)	6.7 (3.9, 11.2)	1.22 (0.59, 2.52)	28.9 (21.8, 37.2)	2.08 (1.47, 2.95)	14.9 (10.5, 20.8)	1.41 (0.90, 2.19)	33.7 (26.1,42.3)	1.74 (1.29, 2.33)
	Some college or associate degree	8.7 (5.9, 12.6)	1.61 (0.92, 2.82)	20.0 (15.8, 24.9)	1.77 (1.26, 2.48)	4.1 (2.5, 6.6)	0.75 (0.38, 1.47)	23.0 (18.5, 28.2)	1.65 (1.21, 2.24)	16.8 (12.9, 21.7)	1.57 (1.08, 2.29)	32.0 (26.9, 37.6)	1.62 (1.27, 2.07)
	College degree or higher	5.2 (3.5,7.5)	ref	11.3 (8.9, 14.2)	ref	4.9 (3.2,7.5)	ref	13.6 (11.0, 16.6)	ref	10.3 (8.0, 13.3)	ref	19.3 (16.3, 22.7)	ref
Insurance Coverage^||^	Any private insurance	6.4 (4.4, 9.3)	ref	12.0 (9.8, 14.7)	ref	4.6 (3.0,6.8)	ref	15.9 (13.1, 19.1)	ref	11.8 (9.5, 14.6)	ref	22.3 (19.2, 25.7)	ref
	Public insurance only	10.0 (7.0, 14.2)	1.03 (0.44, 2.41)	23.3 (18.4, 29.0)	1.62 (1.06, 2.46)	6.8 (4.5, 10.2)	1.03 (0.42, 2.53)	27.2 (21.9, 33.3)	1.33 (0.90, 1.98)	16.5 (12.7, 21.2)	1.37 (0.83, 2.26)	33.9 (27.9, 40.4)	1.28 (0.93, 1.78)
	None	3.0 (1.2, 7.7)	0.33 (0.10, 1.15)	11.6 (5.0, 24.6)	0.84 (0.34, 2.06)	1.0 (0.2, 5.5)	0.17 (0.03, 1.14)	13.4 (6.2, 26.7)	0.68 (0.29, 1.59)	3.6 (1.3, 9.7)	0.31 (0.10, 0.92)	15.8 (7.8, 29.4)	0.61 (0.29, 1.30)
ACE Scores^††^	0	3.1 (2.1, 4.6)	ref	10.7 (8.3, 13.7)	ref	2.8 (1.9, 4.3)	ref	12.1 (9.6, 15.2)	ref	10.9 (8.4, 13.9)	ref	18.2 (15.1, 21.7)	ref
	1	7.8 (4.7, 12.5)	2.24 (1.19, 4.23)	16.2 (12.0, 21.6)	1.45 (0.97, 2.16)	6.3 (3.5, 11.0)	2.04 (0.94, 4.43)	16.3 (12.1, 21.7)	1.20 (0.82, 1.77)	12.6 (8.9, 17.6)	1.07 (0.68, 1.68)	23.7 (18.6, 29.7)	1.22 (0.90, 1.65)
	2–3	9.8 (6.2, 15.2)	2.78 (1.37, 5.66)	19.2 (14.2, 25.5)	1.54 (0.99, 2.40)	7.9 (4.6, 13.4)	2.60 (1.23, 5.49)	26.1 (20.1, 33.1)	1.79 (1.22, 2.62)	19.5 (14.2, 26.1)	1.65 (1.07, 2.54)	33.1 (26.3, 40.7)	1.59 (1.18, 2.15)
	4 or more	17.9 (10.1, 29.8)	4.90 (2.56, 9.40)	27.1 (18.5, 37.9)	2.16 (1.35, 3.46)	5.3 (3.0, 9.2)	1.88 (0.78, 4.49)	41.5 (31.2, 52.6)	2.82 (1.92, 4.14)	8.5 (5.2, 13.8)	0.78 (0.43, 1.44)	44.4 (34.0, 55.3)	2.15 (1.54, 3.02)

ACE = adverse childhood experiences; aPR = adjusted prevaience ratio; autism = autism spectrum disorder; CI = Confidence Interval; FPL = Federal Poverty Level; NH = non-Hispanic.

† Any current autism, developmental delay, intellectual disability, learning disability, and/or speech or other language disorder.

‡ Confidence interval calculated using Taylor Series.

§ No confounders identified for adjustment.

¶ Adjusted for race and ethnicity and highest level of caregiver education.

# Adjusted for race and ethnicity.

|| Adjusted for family income.

†† Adjusted for race and ethnicity, age, family income, primary caregiver marital status, highest level of caregiver education, health insurance coverage.
