# Supplementary material for: Mental, behavioural, and developmental disorders among U.S. Children with and without heart conditions, 2016–2021
Source: Cardiol Young. Author manuscript; Available in PMC 2025 Sep 25. (PMC12462410; doi:10.1017/S1047951125100760)
Supplement: SUP - Dorsey - mental-behavioural-and-developmental-disorders-among-us-children-with-and-without-heart-conditions-2016-2021 [file NIHMS2109311-supplement-SUP_-_Dorsey_-_mental-behavioural-and-developmental-disorders-among-us-children-with-and-without-heart-conditions-2016-2021.docx]

**Supplemental Figure S1: Directed Acyclic Graph (DAG) for Aim 1 of Primary Analyses (Relationship Between Heart Condition Status and Mental, Behavioral, or Developmental Disorders (MBDDs))**


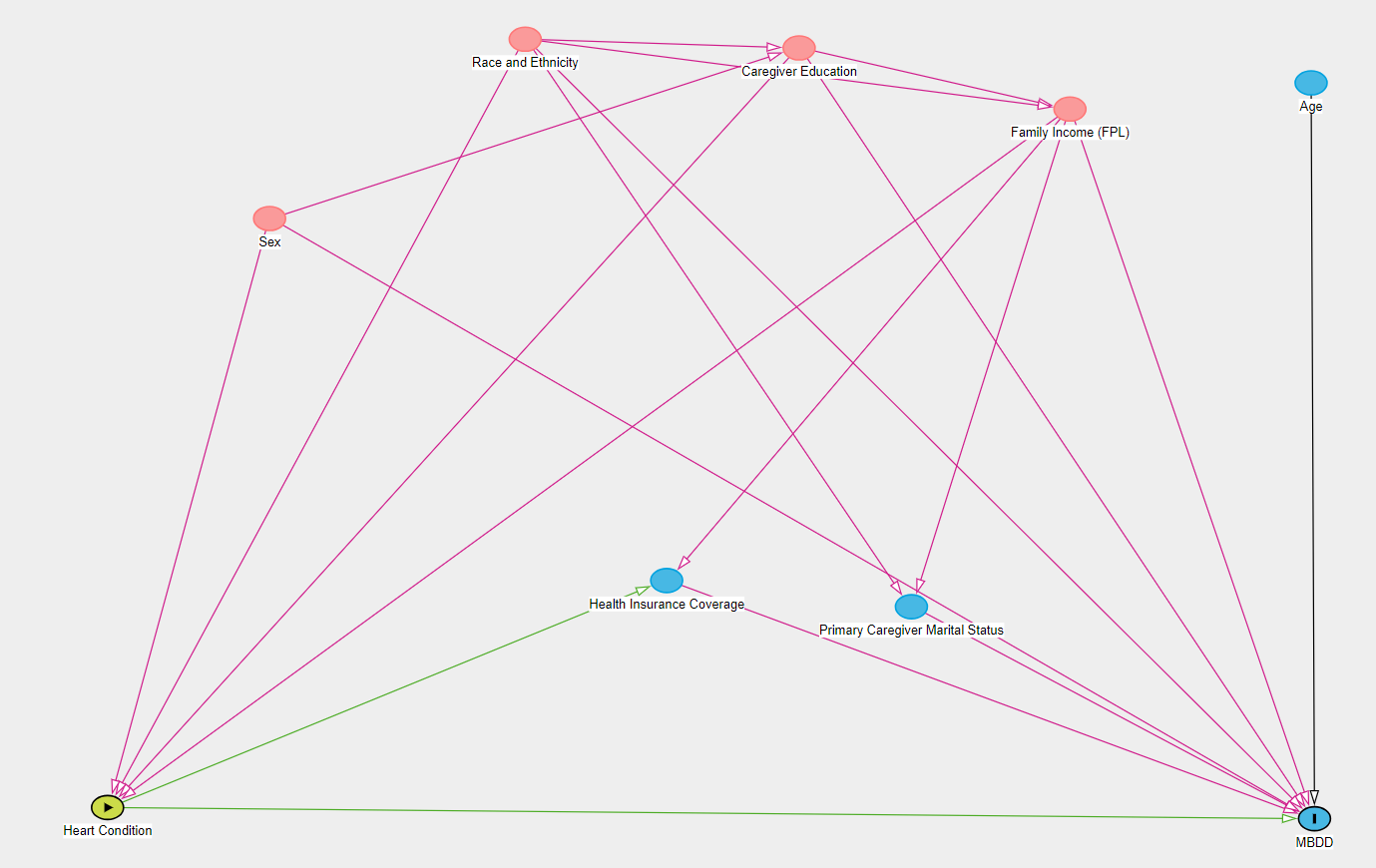


**Supplemental Figure S2: Directed Acyclic Graphs (DAG) for Aim 2 of Primary Analyses (Relationship Between Each Demographic and Contextual Characteristic and Mental, Behavioral, or Developmental Disorders (MBDDs))**

| **Sex**  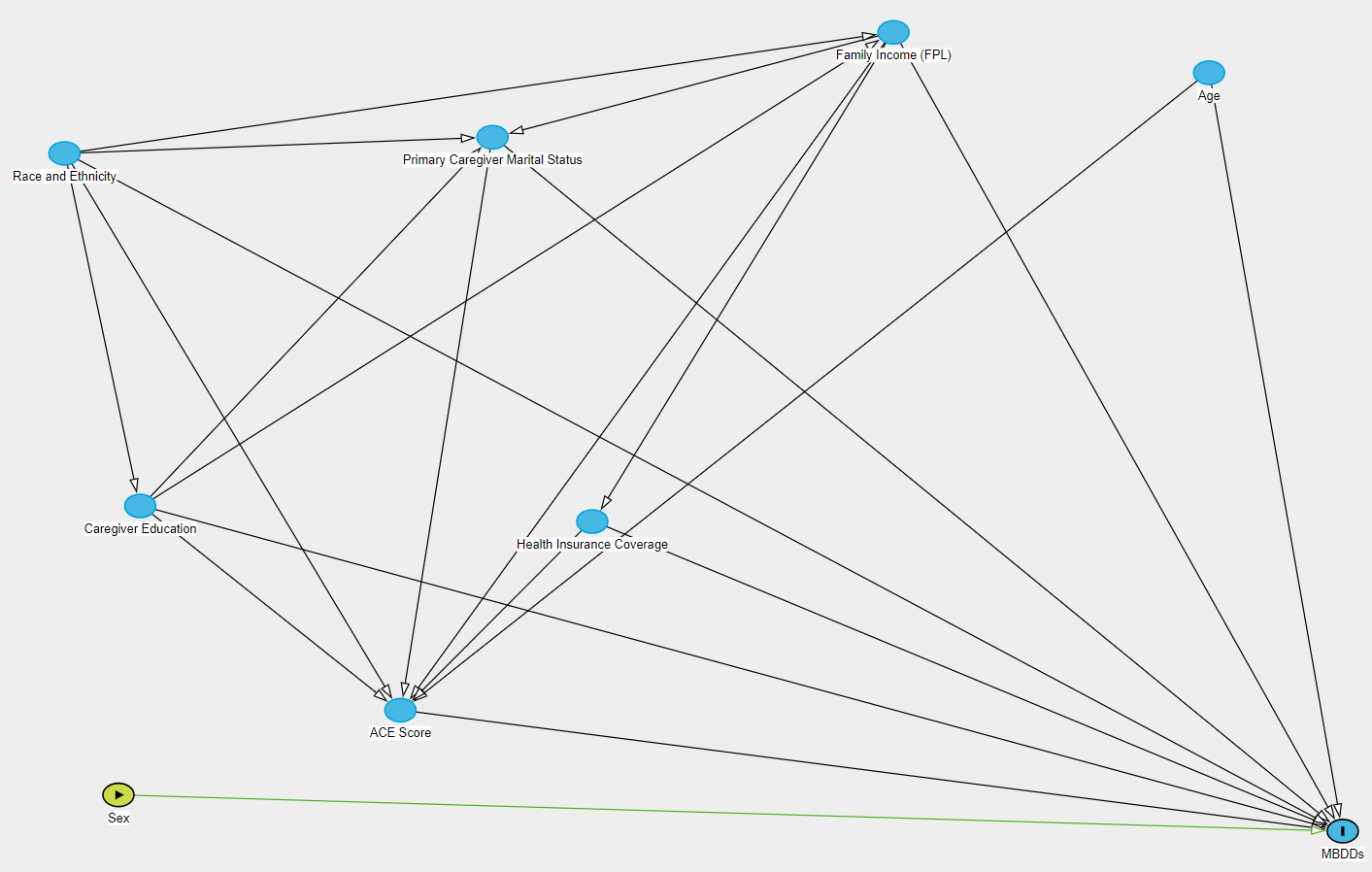 | **Race and Ethnicity**  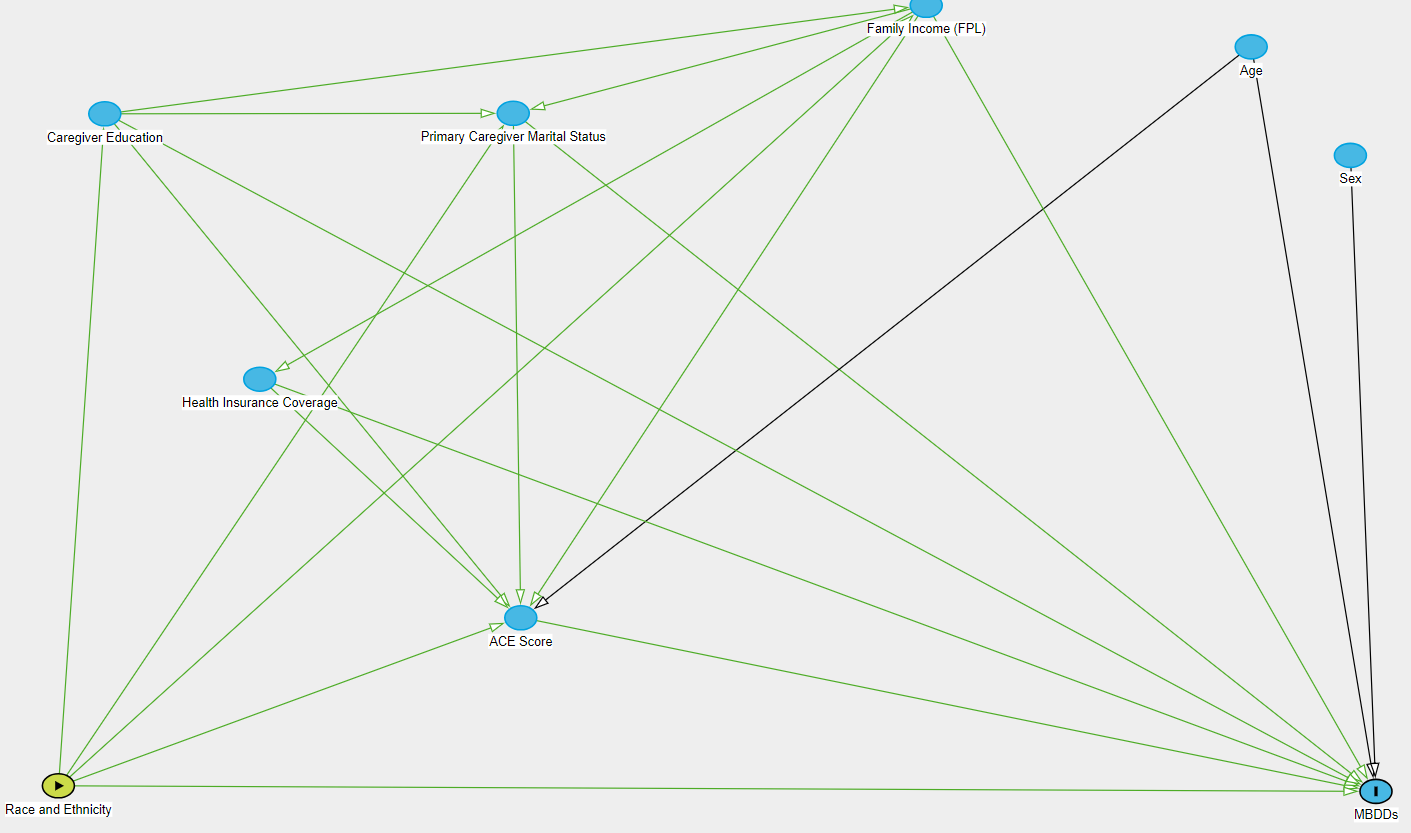 |
| --- | --- |
| **Age**  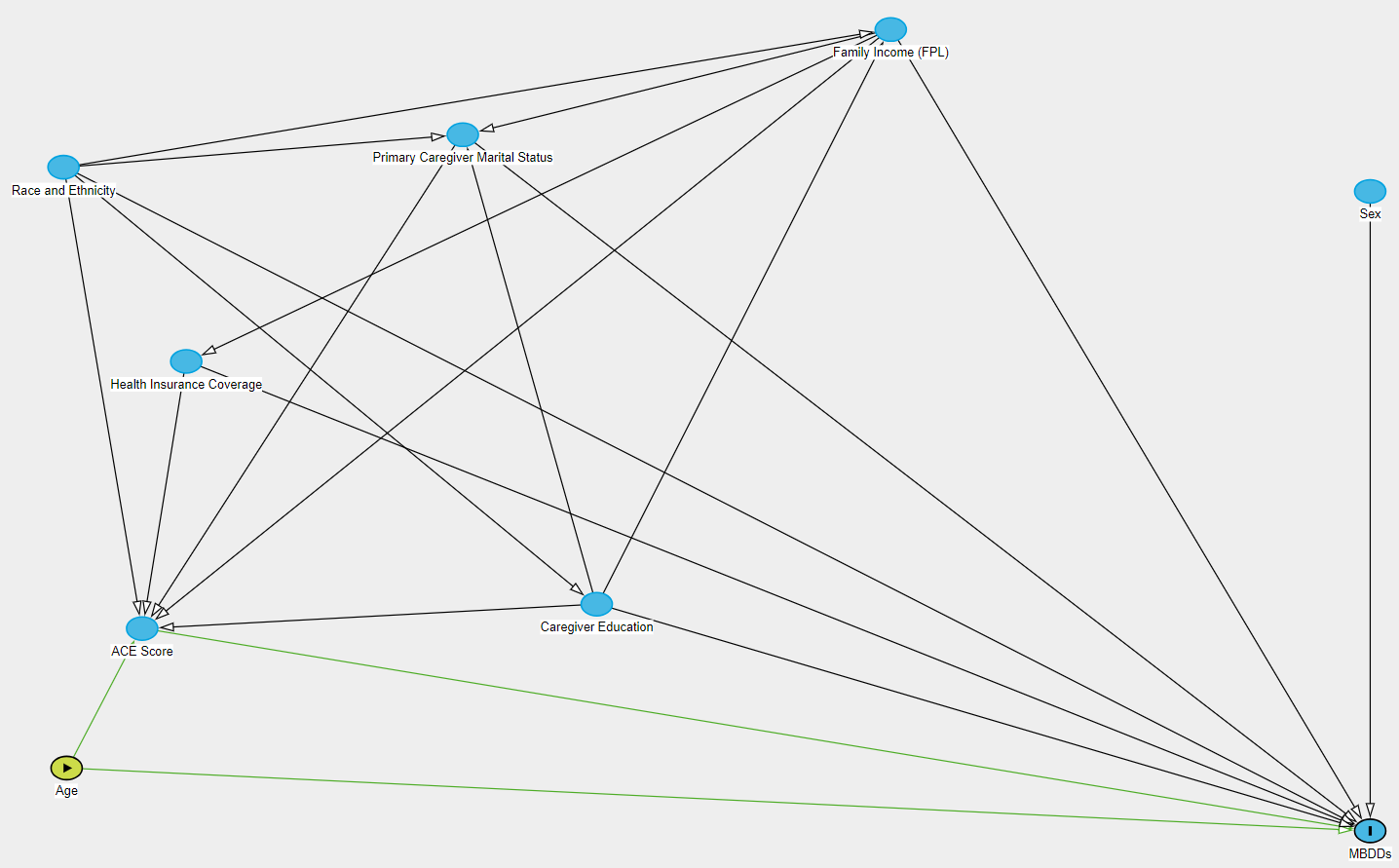 | **Family Income (FPL)**  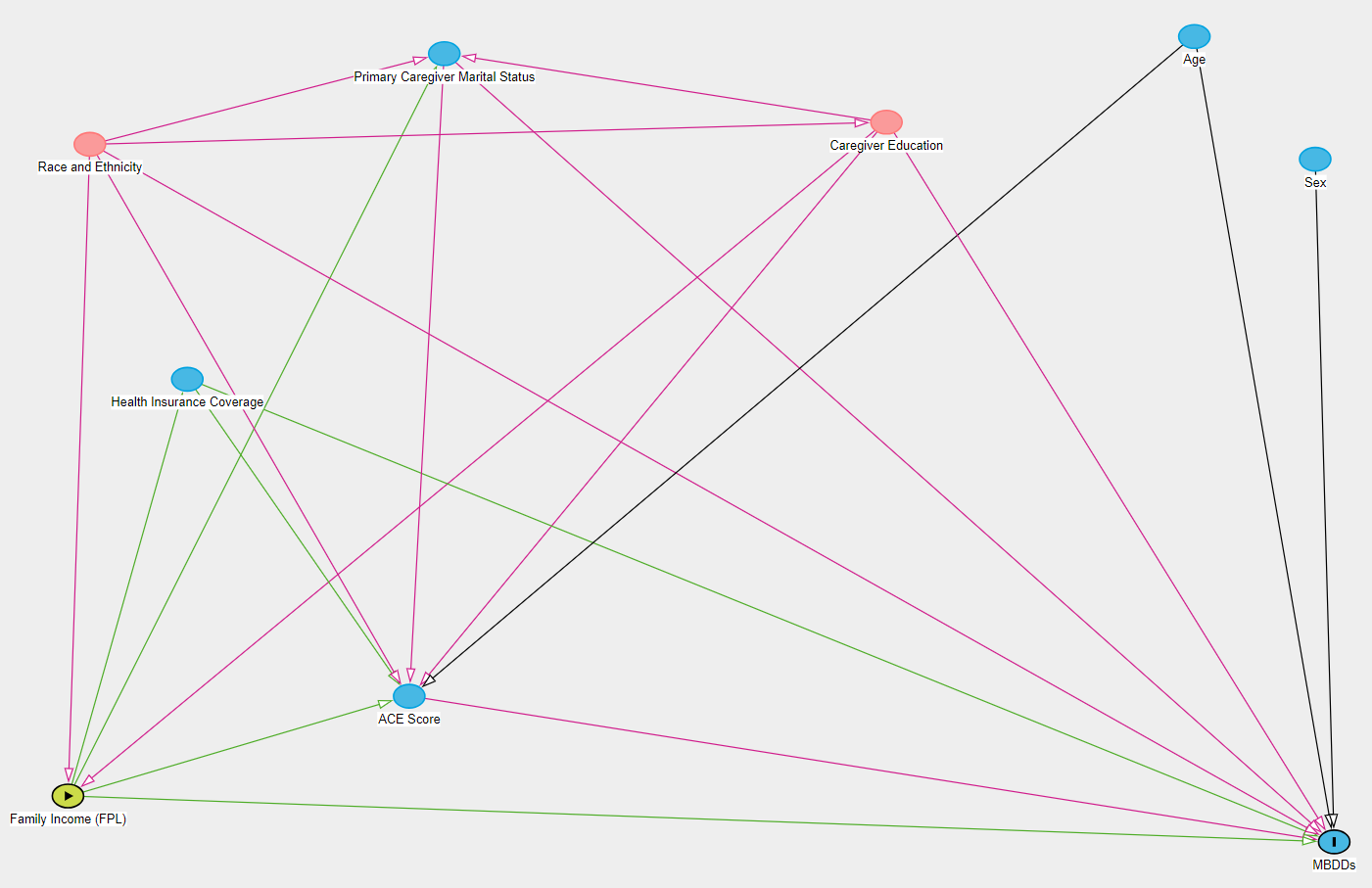 |

| **Primary Caregiver Marital Status**  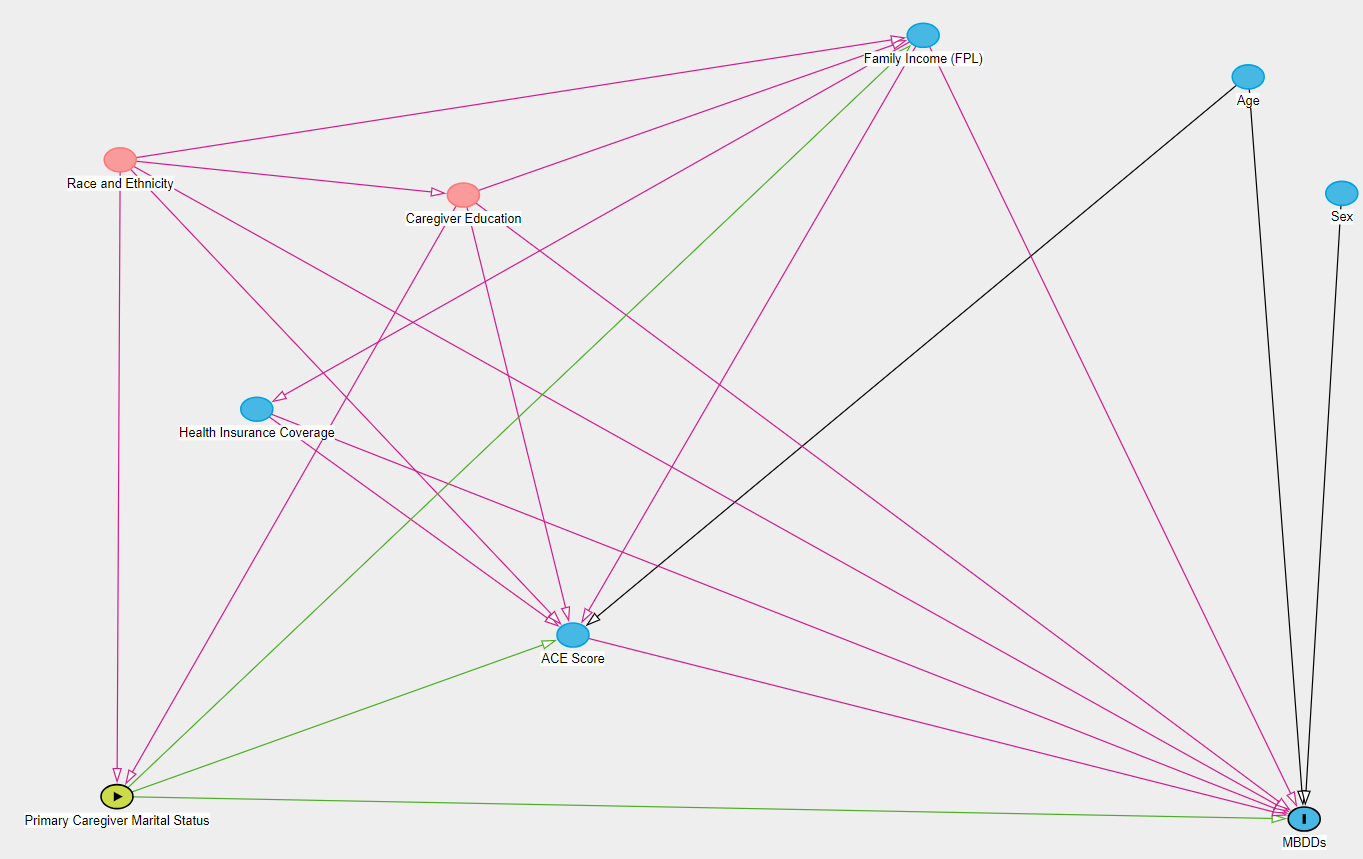 | **Highest Level of Caregiver Education**  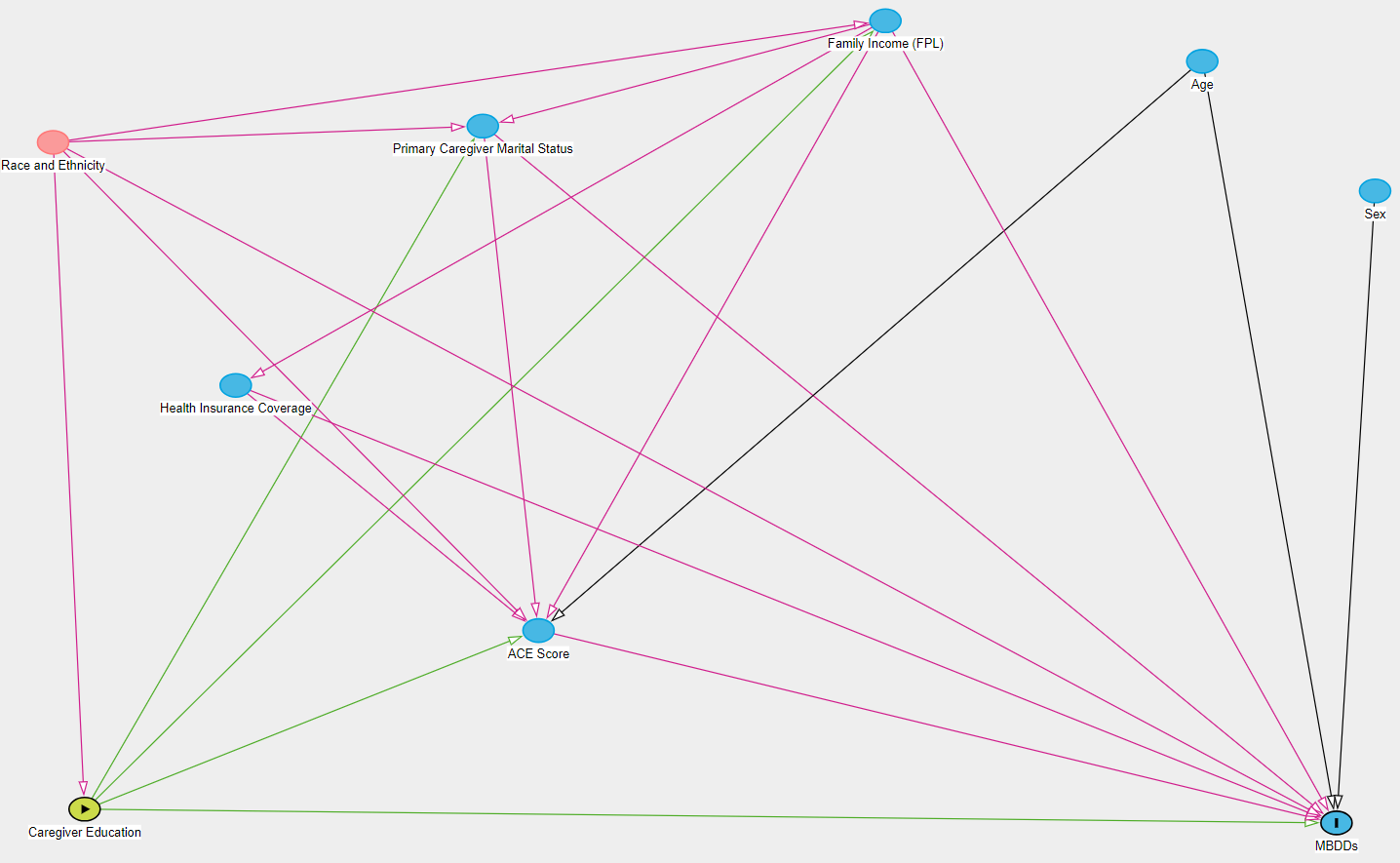 |
| --- | --- |
| **Health Insurance Coverage**  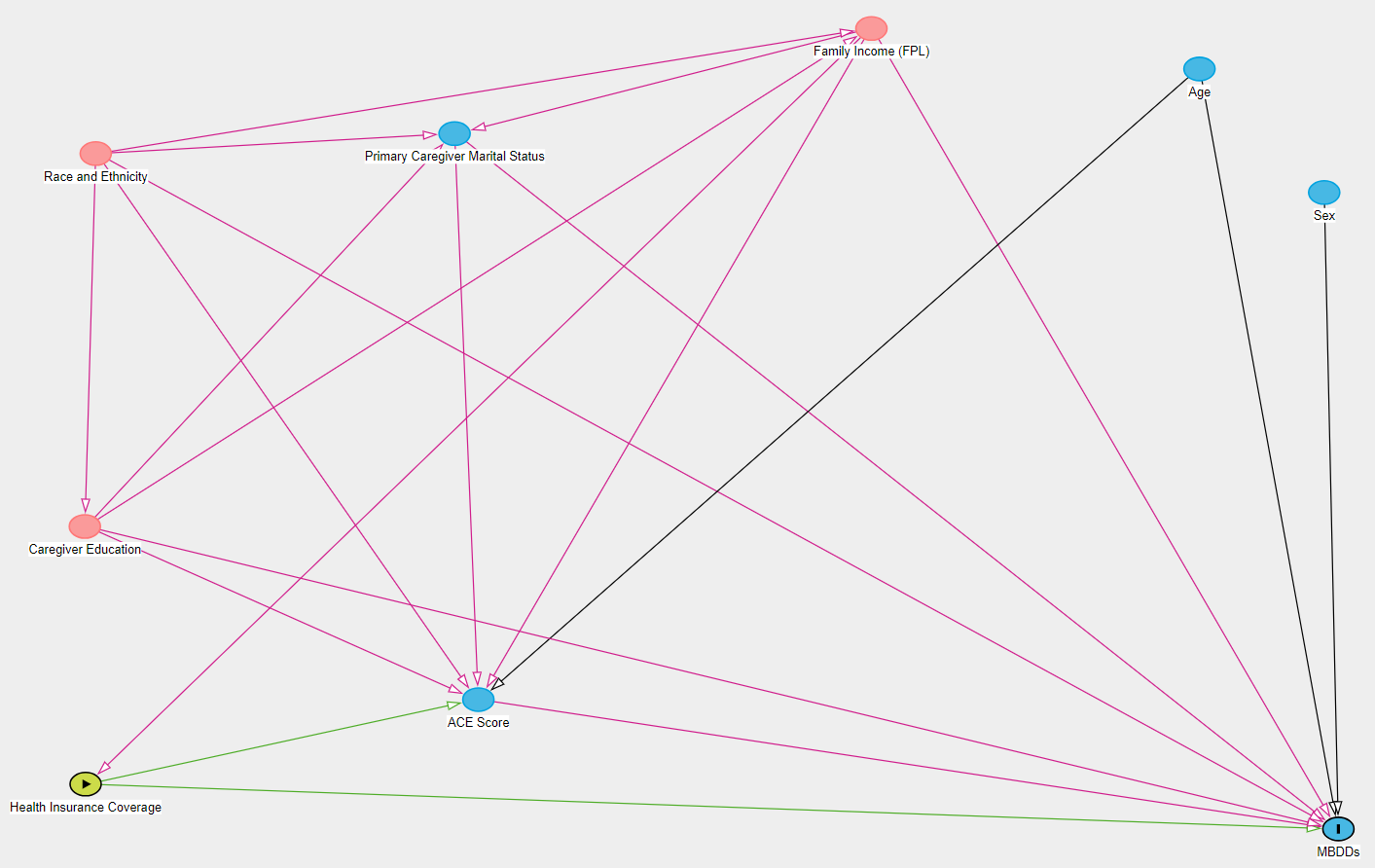 | **ACE Score**  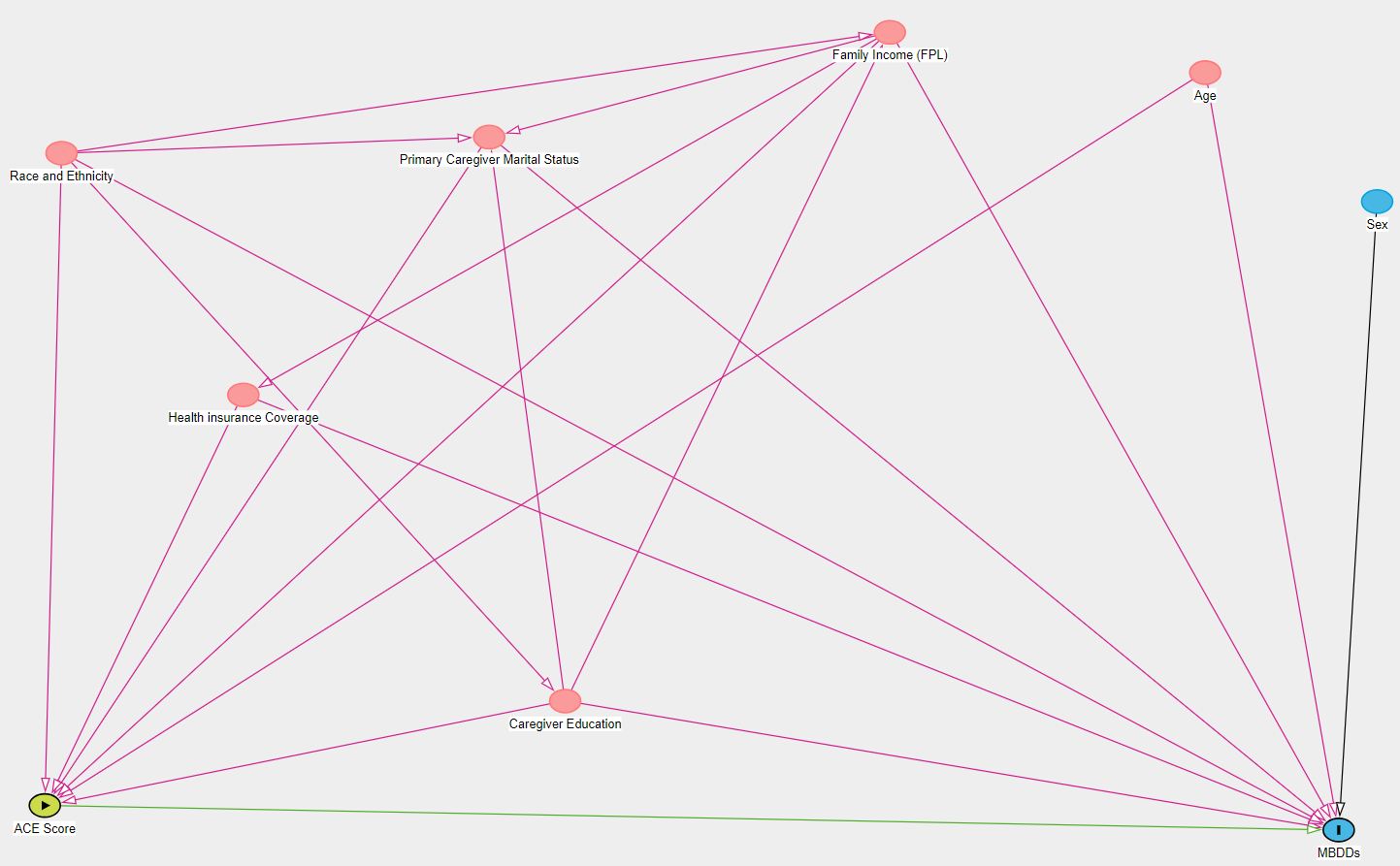 |

## Supplemental Figure S3. Exclusion Criteria and Analytic Sample of US Children 6-17 Years Old without Caregiver-Reported Down Syndrome, National Survey of Children’s Health, United States, 2016-2021


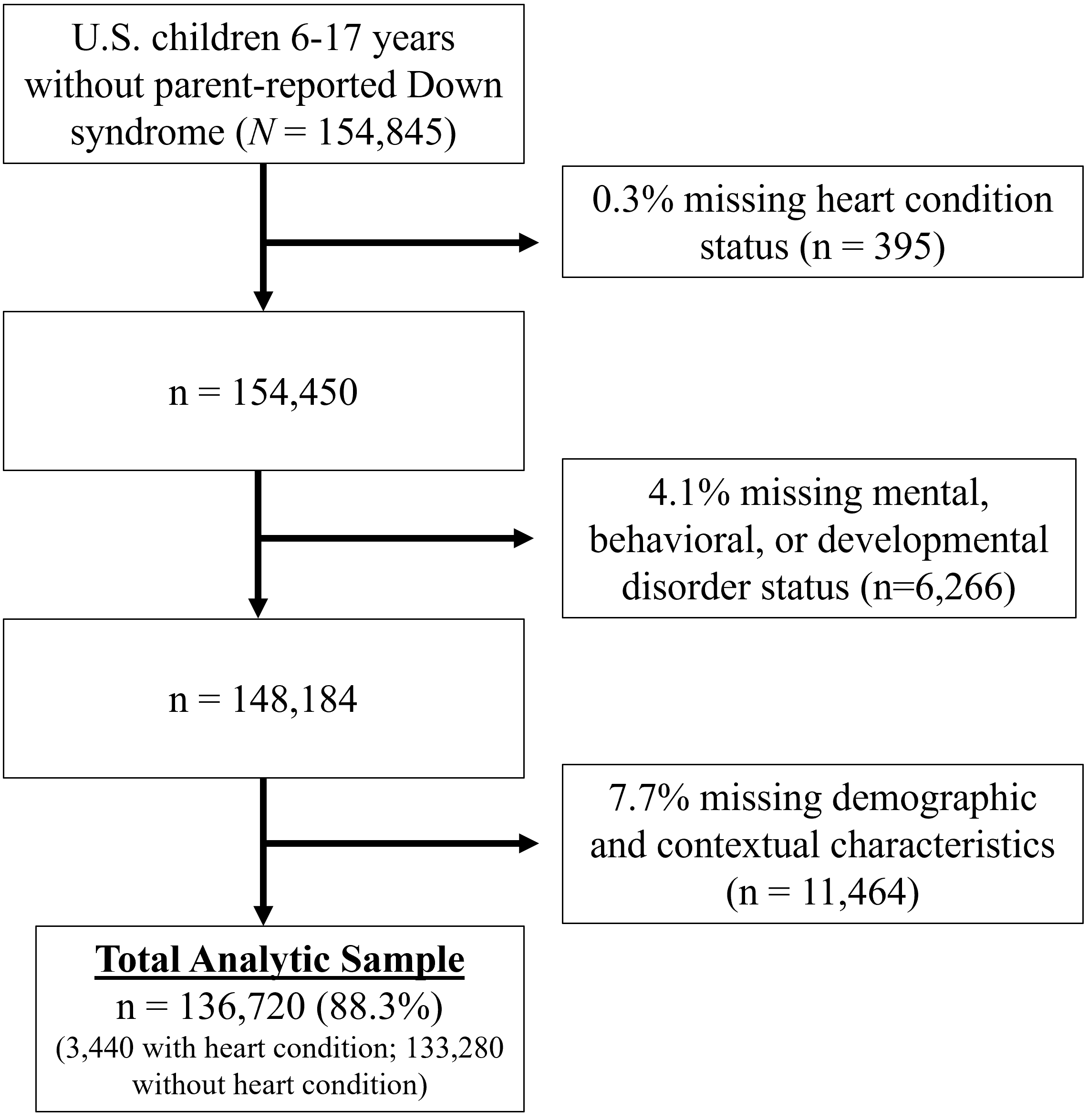


U.S.: United States

Frequencies and percentages in this figure are unweighted

**Supplemental Table S1. Characteristics of Children 6-17 Years Old by Heart Condition Status and Inclusion in the Analytic Sample, National Survey of Children’s Health, United States, 2016-2021**

|  | | **Heart Condition** | | | |  | **No Heart Condition** | | | |  |
| --- | --- | --- | --- | --- | --- | --- | --- | --- | --- | --- | --- |
|  | | **Included** | | **Excluded** | |  | **Included** | | **Excluded** | |  |
| **Characteristic** |  | **Unweighted**  **N** | **Weighted Percent (95% CI**^†^**)** | **Unweighted**  **N** | **Weighted Percent (95% CI**^†^**)** | **Chi-square p-value** | **Unweighted**  **N** | **Weighted Percent (95% CI**^†^**)** | **Unweighted**  **N** | **Weighted Percent (95% CI**^†^**)** | **Chi-square p-value** |
| Sex | Male | 1,891 | 51.2 (47.5, 54.9) | 224 | 64.1 (54.1, 73.0) | 0.02 | 68,788 | 51.2 (50.6, 51.8) | 8,959 | 50.1 (48.4, 51.9) | 0.28 |
|  | Female | 1,549 | 48.8 (45.1, 52.5) | 137 | 35.9 (27.0, 45.9) |  | 64,492 | 48.8 (48.2, 49.4) | 8,410 | 49.9 (48.1, 51.6) |  |
| Race and ethnicity | NH White | 2,480 | 58.1 (54.3, 61.8) | 224 | 42.4 (33.2, 52.1) | 0.01 | 92,166 | 51.7 (51.1, 52.3) | 10,484 | 39.5 (38.0, 41.1) | <0.0001 |
|  | NH Black | 215 | 12.5 (10.3, 15.0) | 42 | 24.4 (16.9, 33.8) |  | 8,180 | 12.9 (12.5, 13.4) | 1,835 | 19.3 (18.0, 20.7) |  |
|  | Hispanic | 370 | 21.3 (17.8, 25.4) | 47 | 21.8 (12.5, 35.2) |  | 15,949 | 25.0 (24.3, 25.6) | 2,618 | 31.2 (29.2, 33.2) |  |
|  | Other | 375 | 8.1 (6.6, 10.0) | 48 | 11.5 (5.7, 21.7) |  | 16,985 | 10.4 (10.1, 10.7) | 2,432 | 10.0 (9.2, 10.8) |  |
| Age (years) | 6-11 years | 1,445 | 49.7 (46.1, 53.4) | 149 | 51.8 (41.6, 61.9) | 0.71 | 57,998 | 50.0 (49.4, 50.6) | 7179 | 46.9 (45.2, 48.7) | 0.002 |
|  | 12-17 years | 1,995 | 50.3 (46.6, 53.9) | 212 | 48.2 (38.1, 58.4) |  | 75,282 | 50.0 (49.4, 50.6) | 10190 | 53.1 (51.3, 54.8) |  |
| Family Income (% FPL) | <100% | 434 | 19.8 (15.9, 24.4) | 68 | 31.0 (20.5, 44.0) | 0.04 | 14,439 | 18.0 (17.5, 18.6) | 3194 | 28.5 (26.4, 30.6) | <0.0001 |
|  | 100-199% | 592 | 20.3 (16.5, 24.6) | 74 | 29.4 (19.8, 41.2) |  | 21,000 | 21.4 (20.8, 21.9) | 3498 | 24.7 (22.9, 26.5) |  |
|  | 200-399% | 1,103 | 31.0 (27.5, 34.6) | 115 | 19.7 (13.3, 28.1) |  | 40,924 | 28.5 (28.0, 29.1) | 4753 | 23.5 (22.0, 25.2) |  |
|  | >400% | 1,312 | 28.9 (25.9, 32.1) | 104 | 19.9 (13.8, 27.7) |  | 56,918 | 32.1 (31.5, 32.6) | 5924 | 23.3 (22.0, 24.7) |  |
| Primary Caregiver Marital Status | Missing |  |  | 73 |  | 0.31 |  |  | 4,279 |  | 0.002 |
|  | Married or living with a partner | 2,715 | 73.4 (69.4, 77.0) | 209 | 65.6 (54.7, 75.1) |  | 107,602 | 77.8 (77.2, 78.3) | 9958 | 74.6 (72.9, 76.3) |  |
|  | Never married | 193 | 8.3 (6.4, 10.9) | 26 | 13.0 (7.5, 21.4) |  | 6,374 | 7.2 (6.8, 7.5) | 860 | 8.3 (7.3, 9.4) |  |
|  | Divorced, separated, or widowed | 532 | 18.3 (15.0, 22.1) | 53 | 21.4 (14.1, 31.2) |  | 19,304 | 15.0 (14.6, 15.5) | 2272 | 17.0 (15.7, 18.5) |  |
| Highest Caregiver Education |  |  |  | 16 |  | 0.003 |  |  | 857 |  | <0.0001 |
|  | High school or less than high school | 509 | 24.9 (21.3, 29.0) | 90 | 49.8 (39.3, 60.3) |  | 20,476 | 28.7 (28.0, 29.4) | 4083 | 42.4 (40.5, 44.3) |  |
|  | Some college or associate degree | 879 | 23.8 (21.2, 26.6) | 86 | 16.4 (11.6, 22.6) |  | 31,057 | 21.8 (21.3, 22.3) | 4062 | 20.7 (19.4, 22.2) |  |
|  | College degree or higher | 2,052 | 51.3 (47.6, 54.9) | 169 | 33.8 (25.5, 43.2) |  | 81,747 | 49.5 (48.9, 50.2) | 8367 | 36.9 (35.3, 38.5) |  |
| Insurance Coverage | Missing |  |  | 47 |  | 0.09 |  |  | 2,491 |  | <0.0001 |
|  | Any private insurance | 2,495 | 60.6 (56.7, 64.4) | 202 | 46.6 (35.8, 57.7) |  | 101,838 | 64.5 (63.8, 65.1) | 9913 | 52.0 (50.0, 53.9) |  |
|  | Public insurance only | 825 | 34.3 (30.6, 38.3) | 90 | 40.1 (28.7, 52.7) |  | 25,584 | 28.8 (28.1, 29.4) | 3915 | 37.3 (35.3, 39.3) |  |
|  | None | 120 | 5.1 (3.4, 7.6) | 22 | 13.3 (6.3, 26.0) |  | 5,858 | 6.8 (6.4, 7.2) | 1050 | 10.7 (9.4, 12.2) |  |
| ACE Scores | Missing |  |  | 190 |  | 0.43 |  |  | 9,133 |  | <0.0001 |
|  | 0 | 1,604 | 40.6 (37.2, 44.1) | 74 | 44.5 (29.1, 61.0) |  | 74,752 | 53.2 (52.6, 53.8) | 3957 | 47.0 (44.5, 49.6) |  |
|  | 1 | 818 | 25.3 (22.1, 28.8) | 39 | 25.1 (14.0, 40.8) |  | 29,982 | 23.9 (23.4, 24.5) | 2000 | 24.2 (22.2, 26.4) |  |
|  | 2-3 | 688 | 23.1 (19.8, 26.6) | 38 | 15.6 (9.3, 25.0) |  | 20,079 | 16.0 (15.6, 16.5) | 1497 | 18.0 (16.2, 20.0) |  |
|  | 4 or more | 330 | 11.0 (9.0, 13.5) | 20 | 14.8 (7.4, 27.2) |  | 8,467 | 6.9 (6.6, 7.2) | 782 | 10.7 (9.1, 12.5) |  |
| Survey Year | 2016 | 718 | 17.5 (14.8, 20.5) | 92 | 25.2 (16.2, 37.1) | 0.50 | 30,324 | 16.3 (15.9, 16.7) | 4379 | 18.4 (17.3, 19.6) | 0.001 |
|  | 2017 | 334 | 16.9 (13.8, 20.6) | 25 | 10.2 (5.1, 19.2) |  | 13,382 | 16.8 (16.3, 17.4) | 1622 | 15.7 (14.2, 17.4) |  |
|  | 2018 | 489 | 15.6 (13.4, 18.0) | 47 | 17.5 (10.6, 27.4) |  | 19,036 | 16.8 (16.4, 17.3) | 2266 | 16.4 (15.1, 17.9) |  |
|  | 2019 | 491 | 15.5 (13.4, 18.0) | 49 | 14.8 (8.9, 23.6) |  | 18,412 | 17.0 (16.5, 17.5) | 2206 | 15.1 (13.9, 16.4) |  |
|  | 2020 | 699 | 16.7 (14.2, 19.6) | 73 | 14.2 (9.8, 20.3) |  | 26,537 | 16.6 (16.2, 17.0) | 3210 | 16.8 (15.5, 18.1) |  |
|  | 2021 | 709 | 17.8 (15.2, 20.7) | 75 | 18.1 (12.5, 25.6) |  | 25,589 | 16.4 (16.0, 16.8) | 3686 | 17.5 (16.4, 18.8) |  |
| Ever had a Special Education or Early Intervention Plan | Missing | 7 |  | 5 |  | 0.74 | 328 |  | 329 |  | 0.18 |
|  | Yes | 1,094 | 30.7 (27.6, 34.0) | 131 | 32.4 (24.0, 42.0) |  | 22,228 | 15.5 (15.1, 15.9) | 3130 | 16.5 (15.2, 17.8) |  |
|  | No | 2,339 | 69.3 (66.0, 72.4) | 225 | 67.6 (58.0, 76.0) |  | 110,724 | 84.5 (84.1, 84.9) | 13910 | 83.5 (82.2, 84.8) |  |
| Took Medication in the Past Year for Emotions, Concentration, or Behavior | Missing | 34 |  | 11 |  | 0.44 | 1,411 |  | 560 |  | 0.002 |
|  | Yes | 621 | 16.5 (14.3, 19.0) | 78 | 19.4 (13.4, 27.2) |  | 15,104 | 9.1 (8.8, 9.4) | 2268 | 10.6 (9.7, 11.5) |  |
|  | No | 2,785 | 83.5 (81.0, 85.7) | 272 | 80.6 (72.8, 86.6) |  | 116,765 | 90.9 (90.6, 91.2) | 14541 | 89.4 (88.5, 90.3) |  |
| Received Treatment or Counseling from a Mental Health Professional | Missing | 8 |  | 7 |  | 0.67 | 398 |  | 338 |  | 0.002 |
|  | Yes | 764 | 20.5 (18.0, 23.3) | 98 | 21.6 (15.4, 29.5) |  | 19,235 | 12.1 (11.7, 12.4) | 2659 | 11.9 (11.0, 12.8) |  |
|  | No, but needed | 142 | 4.2 (3.2, 5.5) | 15 | 7.3 (2.4, 19.7) |  | 3,242 | 2.5 (2.3, 2.7) | 562 | 4.0 (3.3, 4.9) |  |
|  | No, but not needed | 2,526 | 75.3 (72.4, 78.1) | 241 | 71.1 (61.0, 79.5) |  | 110,405 | 85.4 (85.0, 85.8) | 13810 | 84.1 (82.9, 85.2) |  |
| Genetic or Inherited Condition | Missing | 11 |  | 3 |  | 0.38 | 381 |  | 221 |  | 0.78 |
|  | Yes | 525 | 13.5 (11.6, 15.7) | 67 | 17.1 (10.7, 26.3) |  | 5,761 | 3.9 (3.7, 4.1) | 735 | 3.8 (3.2, 4.4) |  |
|  | No | 2,904 | 86.5 (84.3, 88.4) | 291 | 82.9 (73.7, 89.3) |  | 127,138 | 96.1 (95.9, 96.3) | 16413 | 96.2 (95.6, 96.8) |  |
| Depression | Missing |  |  | 16 |  | 0.44 |  |  | 668 |  | 0.34 |
|  | Yes | 382 | 8.7 (7.2, 10.5) | 41 | 12.0 (5.9, 22.7) |  | 7,387 | 4.3 (4.1, 4.6) | 951 | 4.7 (4.0, 5.4) |  |
|  | No | 3,058 | 91.3 (89.5, 92.8) | 304 | 88.0 (77.3, 94.1) |  | 125,893 | 95.7 (95.4, 95.9) | 15750 | 95.3 (94.6, 96.0) |  |
| Anxiety Problems | Missing |  |  | 29 |  | 0.83 |  |  | 1,095 |  | <0.0001 |
|  | Yes | 858 | 22.1 (19.6, 24.9) | 93 | 23.2 (15.4, 33.4) |  | 16,683 | 9.7 (9.4, 10.1) | 1734 | 7.9 (7.1, 8.7) |  |
|  | No | 2,582 | 77.9 (75.1, 80.4) | 239 | 76.8 (66.6, 84.6) |  | 116,597 | 90.3 (89.9, 90.6) | 14540 | 92.1 (91.3, 92.9) |  |
| ADHD | Missing |  |  | 28 |  | 0.48 |  |  | 1,460 |  | 0.15 |
|  | Yes | 709 | 20.4 (17.6, 23.5) | 82 | 23.3 (16.5, 31.9) |  | 15,993 | 10.4 (10.0, 10.7) | 2116 | 11.1 (10.2, 12.0) |  |
|  | No | 2,731 | 79.6 (76.5, 82.4) | 251 | 76.7 (68.1, 83.5) |  | 117,287 | 89.6 (89.3, 90.0) | 13793 | 88.9 (88.0, 89.8) |  |
| Behavioral or Conduct Problems | Missing |  |  | 20 |  | 0.13 |  |  | 740 |  | 0.79 |
|  | Yes | 579 | 15.9 (13.8, 18.4) | 73 | 22.2 (15.3, 31.2) |  | 10,758 | 7.7 (7.4, 8.0) | 1410 | 7.8 (7.0, 8.7) |  |
|  | No | 2,861 | 84.1 (81.6, 86.2) | 268 | 77.8 (68.8, 84.7) |  | 122,522 | 92.3 (92.0, 92.6) | 15219 | 92.2 (91.3, 93.0) |  |
| Tourette Syndrome | Missing |  |  | 8 |  | 1.00 |  |  | 483 |  | 0.60 |
|  | Yes | 25 | 0.7 (0.4, 1.2) | 1 | 0.7 (0.1, 4.6) |  | 389 | 0.2 (0.2, 0.3) | 48 | 0.3 (0.1, 0.8) |  |
|  | No | 3,415 | 99.3 (98.8, 99.6) | 352 | 99.3 (95.4, 99.9) |  | 132,891 | 99.8 (99.7, 99.8) | 16838 | 99.7 (99.2, 99.9) |  |
| ≥1 Mental, Emotional, or Behavioral Disorder^‡^ | Missing |  |  | 51 |  | 0.16 |  |  | 2,753 |  | <0.0001 |
|  | Yes | 1,286 | 34.5 (31.3, 37.9) | 148 | 42.9 (32.2, 54.3) |  | 29,305 | 18.7 (18.3, 19.2) | 3762 | 22.1 (20.6, 23.5) |  |
|  | No | 2,154 | 65.5 (62.1, 68.7) | 162 | 57.1 (45.7, 67.8) |  | 103,975 | 81.3 (80.8, 81.7) | 10,854 | 77.9 (76.5, 79.4) |  |
| Autism | Missing |  |  | 15 |  | 0.54 |  |  | 735 |  | 0.39 |
|  | Yes | 247 | 7.5 (5.8, 9.6) | 32 | 9.3 (5.1, 16.2) |  | 4,105 | 3.0 (2.8, 3.2) | 550 | 2.7 (2.3, 3.3) |  |
|  | No | 3,193 | 92.5 (90.4, 94.2) | 314 | 90.7 (83.8, 94.9) |  | 129,175 | 97.0 (96.8, 97.2) | 16,084 | 97.3 (96.7, 97.7) |  |
| Developmental Delay | Missing |  |  | 21 |  | 0.56 |  |  | 697 |  | 0.33 |
|  | Yes | 533 | 15.9 (13.6, 18.4) | 69 | 18.0 (12.2, 25.8) |  | 6,592 | 4.9 (4.6, 5.1) | 907 | 5.3 (4.5, 6.2) |  |
|  | No | 2,907 | 84.1 (81.6, 86.4) | 271 | 82.0 (74.2, 87.8) |  | 126,688 | 95.1 (94.9, 95.4) | 15,765 | 94.7 (93.8, 95.5) |  |
| Intellectual Disability | Missing |  |  | 12 |  | 0.43 |  |  | 558 |  | 0.09 |
|  | Yes | 161 | 5.2 (3.9, 6.9) | 22 | 3.9 (2.0, 7.5) |  | 1,307 | 1.0 (0.9, 1.2) | 215 | 1.5 (1.1, 2.0) |  |
|  | No | 3,279 | 94.8 (93.1, 96.1) | 327 | 96.1 (92.5, 98.0) |  | 131,973 | 99.0 (98.8, 99.1) | 16,596 | 98.5 (98.0, 98.9) |  |
| Learning Disability | Missing |  |  | 21 |  | 0.65 |  |  | 626 |  | 0.003 |
|  | Yes | 651 | 19.6 (17.1, 22.4) | 78 | 18.0 (12.5, 25.2) |  | 10,523 | 7.6 (7.3, 8.0) | 1,570 | 9.3 (8.3, 10.4) |  |
|  | No | 2,789 | 80.4 (77.6, 82.9) | 262 | 82.0 (74.8, 87.5) |  | 122,757 | 92.4 (92.0, 92.7) | 15,173 | 90.7 (89.6, 91.7) |  |
| Speech or Other Language Disorder | Missing |  |  | 17 |  | 0.38 |  |  | 617 |  | 0.61 |
|  | Yes | 414 | 13.0 (11.0, 15.3) | 55 | 16.5 (10.3, 25.3) |  | 5,822 | 4.7 (4.4, 4.9) | 761 | 4.9 (4.1, 5.9) |  |
|  | No | 3,026 | 87.0 (84.7, 89.0) | 289 | 83.5 (74.7, 89.7) |  | 127,458 | 95.3 (95.1, 95.6) | 15,991 | 95.1 (94.1, 95.9) |  |
| ≥1 Developmental Disorder^§^ | Missing |  |  | 38 |  | 0.03 |  |  | 2,294 |  | <0.0001 |
|  | Yes | 879 | 25.9 (23.1, 28.9) | 120 | 37.7 (28.6, 47.9) |  | 15,473 | 11.3 (10.9, 11.7) | 2,216 | 14.1 (12.9, 15.5) |  |
|  | No | 2,561 | 74.1 (71.1, 76.9) | 203 | 62.3 (52.1, 71.4) |  | 117,807 | 88.7 (88.3, 89.1) | 12,859 | 85.9 (84.5, 87.1) |  |
| ≥1 Mental, Behavioral, or Developmental Disorder^¶^ | Missing |  |  | 67 |  | 0.002 |  |  | 4,131 |  | <0.0001 |
|  | Yes | 1,546 | 42.9 (39.4, 46.5) | 185 | 62.0 (51.6, 71.4) |  | 34,752 | 23.1 (22.6, 23.6) | 4,539 | 30.6 (28.8, 32.4) |  |
|  | No | 1,894 | 57.1 (53.5, 60.6) | 109 | 38.0 (28.6, 48.4) |  | 98,528 | 76.9 (76.4, 77.4) | 8,699 | 69.4 (67.6, 71.2) |  |

ACE: adverse childhood experiences; aPR: adjusted prevalence ratio; autism: autism spectrum disorder; CI: Confidence Interval; FPL: Federal Poverty Level; NH: non-Hispanic

^†^Confidence interval calculated using Taylor Series

^‡^Any current depression, anxiety problems, ADHD, behavioral or conduct problems, or Tourette syndrome

^§^Any current autism, developmental delay, intellectual disability, learning disability and/or speech or other language disorder

^¶^Any current depression, anxiety problems, ADHD, behavioral or conduct problems, Tourette syndrome, autism, developmental delay, intellectual disability, learning disability, and/or speech or other language disorder

**Supplemental Table S2. Prevalence of Mental, Behavioral, or Developmental Disorders Among Children 6-17 Years Old with and Without Heart Conditions, National Survey of Children’s Health, United States, 2016-2021** **(Sensitivity Analysis Excluding children with Any Genetic Condition in Addition to Down Syndrome)**

|  | **Heart Condition** | | **No Heart Condition** | |  |
| --- | --- | --- | --- | --- | --- |
| **Characteristic** | **N** | **Weighted Percent (95% CI**^†^**)** | **N** | **Weighted Percent (95% CI**^†^**)** | **aPR (Heart Condition v. No Heart Condition)**^‡^  **(95% CI)** |
| ≥1 Mental, Behavioral, or Developmental Disorder^§^ | 1,144 | 37.0 (33.3,40.8) | 31,015 | 21.5 (21.0,22.0) | 1.68  (1.52, 1.87) |
| Depression | 272 | 6.9 (5.4,8.6) | 6,545 | 4.0 (3.7,4.2) | 1.68  (1.31, 2.14) |
| Anxiety Problems | 624 | 18.5 (16.0,21.3) | 14,758 | 8.9 (8.6,9.2) | 2.00  (1.72, 2.32) |
| ADHD | 530 | 17.5 (14.7,20.8) | 13,907 | 9.3 (9.0,9.7) | 1.83  (1.52, 2.19) |
| Behavioral or Conduct Problems | 392 | 12.2 (10.3,14.5) | 9,016 | 6.8 (6.5,7.1) | 1.76  (1.49, 2.08) |
| Tourette syndrome | 14 | 0.5 (0.2,1.1) | 337 | 0.2 (0.2,0.3) | 2.24  (0.96, 5.24) |
| ≥1 Mental, Emotional, or Behavioral Disorder^¶^ | 959 | 29.4 (26.1,33.0) | 26,112 | 17.4 (16.9,17.8) | 1.65  (1.46, 1.87) |
| Autism | 145 | 5.4 (3.9,7.5) | 3,180 | 2.4 (2.2,2.7) | 2.23  (1.61, 3.08) |
| Developmental Delay | 297 | 11.5 (9.3,14.1) | 5,104 | 4.0 (3.8,4.3) | 2.84  (2.30, 3.49) |
| Intellectual Disability | 61 | 2.7 (1.6,4.4) | 843 | 0.7 (0.6,0.9) | 3.85  (2.29, 6.47) |
| Learning Disability | 393 | 14.9 (12.5,17.7) | 8,689 | 6.6 (6.3,7.0) | 2.22  (1.86, 2.64) |
| Speech or Other Language Disorder | 245 | 10.2 (8.3,12.6) | 4,743 | 4.1 (3.8,4.3) | 2.49  (2.01, 3.10) |
| ≥1 Developmental Disorder**^#^** | 571 | 20.6 (17.8,23.6) | 13,030 | 10.0 (9.6,10.4) | 2.02  (1.76, 2.32) |

aPR: adjusted prevalence ratio; autism: autism spectrum disorder; CI: confidence interval

^†^Confidence interval calculated using Taylor Series

^‡^Adjusted for child’s sex, race and ethnicity, income, and the highest level of education in the household

^§^Any current depression, anxiety problems, ADHD, behavioral or conduct problems, Tourette syndrome, autism, developmental delay, intellectual disability, learning disability and/or speech or other language disorder

^¶^Any current depression, anxiety problems, ADHD, behavioral or conduct problems, and/or Tourette syndrome

**^#^**Any current autism, developmental delay, intellectual disability, learning disability and/or speech or other language disorder

**Supplemental Table S3. Demographic and Contextual Characteristics Associated with Mental, Emotional, or Behavioral Disorders Among Children 6-17 Years Old with Heart Conditions, National Survey of Children’s Health, United States, 2016-2021 (Sensitivity Analysis Excluding children with Any Genetic Condition in Addition to Down Syndrome)**

|  | | **Depression** | | **Anxiety Problems** | | **ADHD** | | **Behavioral or Conduct Problems** | | **≥ 1 Mental, Emotional, or Behavioral Disorder**^†^ | | |
| --- | --- | --- | --- | --- | --- | --- | --- | --- | --- | --- | --- | --- |
| **Characteristic** |  | **Weighted Percent (95% CI**^‡^**)** | **aPR**  **(95% CI)** | **Weighted Percent (95% CI**^‡^**)** | **aPR**  **(95% CI)** | **Weighted Percent**  **(95% CI**^‡^**)** | **aPR**  **(95% CI)** | **Weighted Percent (95% CI**^‡^**)** | **aPR**  **(95% CI)** | **Weighted Percent (95% CI**^‡^**)** | **aPR**  **(95% CI)** |  |
| Sex^§^ | Male | 8.4 (6.1, 11.5) | 1.59  (1.03, 2.47) | 20.1 (16.6, 24.1) | 1.20  (0.90, 1.60) | 22.4 (18.9, 26.5) | 1.81  (1.18, 2.77) | 16.7 (13.7, 20.2) | 2.20  (1.54, 3.14) | 33.9 (29.8,38.4) | 1.37  (1.07, 1.77) |  |
|  | Female | 5.3 (3.9, 7.1) | ref | 16.8 (13.4, 20.7) | ref | 12.4 (8.3, 18.2) | ref | 7.6 (5.6, 10.2) | ref | 24.7 (19.7,30.5) | ref |  |
| Race/ ethnicity^§^ | NH White | 7.5 (5.7, 9.9) | ref | 20.3 (17.3, 23.8) | ref | 17.3 (14.5, 20.7) | ref | 12.5 (10.2, 15.2) | ref | 30.7 (27.0,34.6) | ref |  |
|  | NH Black | 6.4 (3.2, 12.6) | 0.85  (0.40, 1.80) | 24.3 (16.2, 34.9) | 1.19  (0.79, 1.81) | 26.7 (18.4, 37.1) | 1.54  (1.04, 2.29) | 17.9 (11.6, 26.5) | 1.43  (0.90, 2.26) | 36.6 (27.2,47.2) | 1.19  (0.88, 1.62) |  |
|  | Hispanic | 4.1 (1.7, 9.3) | 0.54  (0.22, 1.32) | 11.0 (6.4, 18.2) | 0.54  (0.31, 0.93) | 13.5 (6.2, 26.7) | 0.78  (0.36, 1.66) | 7.5 (4.0, 13.4) | 0.60  (0.32, 1.13) | 23.7 (14.8,35.5) | 0.77  (0.49, 1.22) |  |
|  | Other | 10.2 (5.9, 17.0) | 1.36  (0.75, 2.47) | 16.2 (10.6, 24.1) | 0.80  (0.51, 1.24) | 16.2 (10.1, 24.8) | 0.93  (0.58, 1.51) | 14.9 (9.1, 23.2) | 1.19  (0.71, 1.98) | 25.1 (17.6,34.4) | 0.82  (0.57, 1.17) |  |
| Age (years)^§^ | 6-11 years | 2.4 (1.4, 4.1) | ref | 13.3 (10.3, 17.1) | ref | 14.5 (11.4, 18.3) | ref | 13.7 (10.8, 17.2) | ref | 23.4 (19.4, 27.9) | ref |  |
|  | 12-17 years | 11.5 (8.9, 14.7) | 4.85  (2.63, 8.95) | 23.8 (20.1, 28.1) | 1.79  (1.32, 2.42) | 20.7 (16.1, 26.1) | 1.43  (1.02, 2.00) | 10.7 (8.4, 13.7) | 0.79  (0.56, 1.10) | 35.7 (30.8, 41.0) | 1.53  (1.21, 1.93) |  |
| Family Income (% FPL) ^¶^ | <100% | 8.5 (4.5, 15.6) | 1.73  (0.83, 3.62) | 18.6 (12.1, 27.4) | 1.03  (0.55, 1.93) | 20.7 (13.5, 30.4) | 1.31  (0.72, 2.39) | 15.4 (10.0, 22.9) | 1.23  (0.63, 2.39) | 28.9 (20.2, 39.6) | 1.04  (0.65, 1.66) |  |
|  | 100-199% | 10.3 (6.3, 16.2) | 2.21  (1.18, 4.16) | 21.0 (15.2, 28.3) | 1.26  (0.77, 2.03) | 17.4 (12.3, 23.9) | 1.14  (0.70, 1.85) | 17.7 (12.4, 24.6) | 1.56  (0.87, 2.80) | 32.0 (24.9,40.1) | 1.19  (0.85, 1.66) |  |
|  | 200-399% | 5.8 (3.7, 8.9) | 1.24  (0.72, 2.13) | 18.9 (14.3, 24.5) | 1.17  (0.78, 1.75) | 18.2 (12.0, 26.7) | 1.22  (0.72, 2.06) | 10.2 (7.3, 14.1) | 1.01  (0.62, 1.65) | 30.8 (23.8, 38.8) | 1.15  (0.82, 1.62) |  |
|  | ≥400% | 4.6 (3.3, 6.5) | ref | 16.3 (12.6, 20.7) | ref | 14.9 (11.4, 19.2) | ref | 8.6 (6.0, 12.3) | ref | 26.6 (22.0, 31.8) | ref |  |
| Primary Caregiver Marital Status^¶^ | Married or living with a partner | 5.4 (4.1, 7.2) | ref | 17.0 (14.4, 20.0) | ref | 15.2 (12.7, 18.0) | ref | 11.1 (9.1, 13.5) | ref | 26.1 (23.0, 29.5) | ref |  |
|  | Never married | 5.8 (3.0, 10.9) | 0.91  (0.39, 2.10) | 20.3 (11.0, 34.5) | 1.07  (0.58, 1.97) | 17.8 (9.2, 31.5) | 1.01  (0.52, 1.97) | 15.8 (9.2, 25.6) | 1.03  (0.57, 1.89) | 34.3 (22.2, 48.9) | 1.27  (0.84, 1.93) |  |
|  | Divorced, separated, or widowed | 13.4 (8.2, 21.1) | 2.28  (1.35, 3.86) | 23.6 (16.4, 32.7) | 1.31  (0.90, 1.91) | 27.4 (17.1, 40.8) | 1.76  (1.05, 2.96) | 15.5 (10.1, 23.0) | 1.22  (0.78, 1.91) | 41.0 (29.3, 53.9) | 1.56  (1.09, 2.21) |  |
| Highest Level of Caregiver Education^#^ | High school or less than high school | 8.9 (5.1, 15.0) | 1.83  (1.00, 3.35) | 21.0 (15.0, 28.7) | 1.34  (0.91, 1.97) | 17.3 (12.0, 24.2) | 1.08  (0.65, 1.78) | 15.4 (10.5, 22.1) | 2.05  (1.33, 3.16) | 28.7 (21.5, 37.1) | 1.07  (0.76, 1.51) |  |
|  | Some college or Associate Degree | 8.3 (5.6, 12.0) | 1.68  (1.03, 2.73) | 20.0 (15.6, 25.1) | 1.21  (0.87, 1.66) | 21.1 (16.5, 26.6) | 1.26  (0.83, 1.91) | 18.7 (14.4, 24.0) | 2.39  (1.69, 3.39) | 34.8 (29.0, 41.0) | 1.26  (0.97, 1.63) |  |
|  | College degree or higher | 5.2 (4.0, 6.9) | ref | 16.6 (13.5, 20.1) | ref | 16.1 (12.0, 21.3) | ref | 7.9 (6.3, 9.8) | ref | 27.4 (22.9, 32.5) | ref |  |
| Health Insurance Coverage**^\|\|^** | Any private insurance | 5.5 (4.2, 7.2) | Ref | 17.4 (14.6, 20.7) | ref | 15.7 (12.1, 20.2) | ref | 8.2 (6.6, 10.2) | ref | 27.2 (23.3, 31.6) | ref |  |
|  | Public insurance only | 9.9 (6.6, 14.5) | 1.35  (0.81, 2.25) | 20.8 (15.9, 26.8) | 1.16  (0.74, 1.83) | 21.5 (16.5, 27.5) | 1.37  (0.85, 2.22) | 20.3 (15.5, 26.1) | 2.44  (1.46, 4.07) | 34.5 (27.9, 41.8) | 1.35  (0.97, 1.88) |  |
|  | None | 3.3 (1.4, 7.7) | 0.48  (0.18, 1.25) | 15.7 (7.8, 28.9) | 0.88  (0.41, 1.88) | 13.1 (5.8, 27.2) | 0.83  (0.35, 1.98) | 7.7 (2.9, 18.9) | 0.94  (0.32, 2.70) | 22.7 (12.2, 38.5) | 0.89  (0.48, 1.64) |  |
| ACE Scores^††^ | 0 | 2.6 (1.7, 3.8) | ref | 9.7 (7.6, 12.4) | ref | 8.6 (6.6, 11.1) | ref | 6.5 (4.7, 9.0) | Ref | 17.3 (14.3, 20.7) | ref |  |
|  | 1 | 5.0 (3.3, 7.5) | 1.66  (0.88, 3.13) | 18.3 (13.3, 24.8) | 2.06  (1.35, 3.14) | 19.5 (12.2, 29.5) | 2.26  (1.45, 3.53) | 9.4 (6.5, 13.5) | 1.38  (0.86, 2.20) | 31.2 (23.3, 40.3) | 1.85  (1.36, 2.50) |  |
|  | 2-3 | 10.1 (6.4, 15.8) | 3.05  (1.60, 5.82) | 26.1 (19.6, 33.7) | 2.95  (2.02, 4.32) | 22.9 (17.1, 30.0) | 2.58  (1.66, 4.00) | 18.3 (13.1, 24.9) | 2.28  (1.37, 3.78) | 37.6 (29.6, 46.2) | 2.18  (1.60, 2.95) |  |
|  | 4 or more | 23.2 (14.9, 34.3) | 6.89  (3.88, 12.23) | 39.9 (29.3, 51.7) | 4.79  (3.25, 7.07) | 39.5 (28.7, 51.4) | 4.80  (3.13, 7.34) | 31.1 (22.1, 41.7) | 3.91  (2.39, 6.41) | 59.7 (47.1, 71.2) | 3.56  (2.62, 4.82) |  |

ACE: adverse childhood experiences; aPR: adjusted prevalence ratio; CI: Confidence Interval; FPL: Federal Poverty Level; NH: non-Hispanic

^†^Any current depression, anxiety problems, ADHD, behavioral or conduct problem and/or Tourette syndrome

^‡^Confidence interval calculated using Taylor Series

^§^No confounders identified for adjustment

^¶^Adjusted for race and ethnicity and highest level of caregiver education

^#^Adjusted for race and ethnicity

**^||^**Adjusted for family income

^††^Adjusted for race and ethnicity, age, family income, primary caregiver marital status, highest level of caregiver education, health insurance coverage

**Supplemental Table S4. Demographic and Contextual Characteristics Associated with Developmental Disorders Among Children 6-17 Years Old with Heart Conditions, National Survey of Children’s Health, United States, 2016-2021 (Sensitivity Analysis Excluding children with Any Genetic Condition in Addition to Down Syndrome)**

|  | | **Autism** | | **Developmental Delay** | | **Intellectual Disability** | | **Learning Disability** | | **Speech or Other Language Disorder** | | **≥1 Developmental Disorder**^†^ | |
| --- | --- | --- | --- | --- | --- | --- | --- | --- | --- | --- | --- | --- | --- |
| **Characteristic** |  | **Weighted Percent (95% CI**^‡^**)** | **aPR**  **(95% CI)** | **Weighted Percent (95% CI**^‡^**)** | **aPR (95% CI)** | **Weighted Percent (95% CI**^‡^**)** | **aPR (95% CI)** | **Weighted Percent (95% CI**^‡^**)** | **aPR (95% CI)** | **Weighted Percent (95% CI**^‡^**)** | **aPR (95% CI)** | **Weighted Percent (95% CI**^‡^**)** | **aPR (95% CI)** |
| Sex^§^ | Male | 9.8 (7.0, 13.5) | 10.35  (3.84, 27.86) | 16.0 (12.7, 20.0) | 2.37  (1.49, 3.77) | 4.1 (2.2, 7.2) | 3.37  (1.18, 9.64) | 19.0 (15.4, 23.3) | 1.79  (1.25, 2.56) | 14.4 (11.2, 18.3) | 2.43  (1.56, 3.77) | 27.6 (23.5, 32.0) | 2.07  (1.54, 2.79) |
|  | Female | 0.9 (0.4, 2.4) | ref | 6.7 (4.5, 10.0) | ref | 1.2 (0.5, 2.9) | ref | 10.6 (7.9, 14.1) | ref | 5.9 (4.1, 8.5) | ref | 13.3 (10.3, 17.1) | ref |
| Race/ ethnicity^§^ | NH White | 5.0 (3.3, 7.6) | ref | 10.5 (8.0, 13.8) | ref | 1.4 (0.7, 2.9) | ref | 13.3 (10.5, 16.7) | ref | 8.5 (6.4, 11.1) | ref | 19.1 (15.9, 22.7) | ref |
|  | NH Black | 8.9 (4.6, 16.6) | 1.78  (0.82, 3.87) | 19.6 (12.5, 29.4) | 1.87  (1.12, 3.10) | 7.8 (3.3, 17.0) | 5.50  (1.84, 16.47) | 21.8 (14.6, 31.2) | 1.64  (1.05, 2.56) | 20.1 (12.6, 30.4) | 2.36  (1.41, 3.97) | 32.5 (23.6, 42.8) | 1.70  (1.20, 2.40) |
|  | Hispanic | 4.8 (1.8, 11.8) | 0.96  (0.34, 2.67) | 8.5 (4.5, 15.3) | 0.81  (0.41, 1.58) | 2.7 (0.6, 10.9) | 1.94  (0.39, 9.68) | 14.1 (8.7, 22.0) | 1.06  (0.63, 1.79) | 8.4 (4.5, 15.1) | 0.98  (0.50, 1.92) | 17.1 (11.2, 25.3) | 0.90  (0.57, 1.40) |
|  | Other | 5.2 (2.1, 12.4) | 1.05  (0.39, 2.82) | 14.3 (8.4, 23.2) | 1.36  (0.76, 2.42) | 3.8 (1.5, 9.7) | 2.73  (0.82, 9.06) | 18.8 (11.9, 28.4) | 1.41  (0.86, 2.32) | 13.1 (7.4, 22.0) | 1.54  (0.83, 2.83) | 22.9 (15.3, 32.8) | 1.20  (0.79, 1.83) |
| Age (years)^§^ | 6-11 years | 3.9 (2.4, 6.1) | ref | 12.5 (9.6, 16.1) | ref | 1.9 (0.9, 4.2) | ref | 13.2 (10.3, 16.9) | ref | 12.6 (9.6, 16.3) | ref | 21.2 (17.3, 25.6) | ref |
|  | 12-17 years | 7.1 (4.6, 10.8) | 1.84  (0.98, 3.48) | 10.4 (7.4, 14.4) | 0.83  (0.54, 1.27) | 3.4 (1.8, 6.5) | 1.80  (0.65, 5.04) | 16.7 (13.0, 21.0) | 1.26  (0.89, 1.78) | 7.7 (5.4, 11.0) | 0.61  (0.39, 0.96) | 20.0 (16.2, 24.4) | 0.95  (0.71, 1.25) |
| Family Income (% FPL)^¶^ | <100% | 7.5 (3.6, 14.8) | 1.52  (0.46, 5.05) | 14.8 (8.4, 24.6) | 1.19  (0.50, 2.81) | 2.9 (1.0, 8.0) | 3.09  (0.38, 25.08) | 19.0 (12.0, 28.6) | 1.09  (0.56, 2.11) | 7.0 (3.7, 12.7) | 0.46  (0.17, 1.22) | 22.3 (14.6, 32.6) | 0.93  (0.51, 1.70) |
|  | 100-199% | 6.6 (3.4, 12.4) | 1.44  (0.49, 4.27) | 17.0 (11.6, 24.2) | 1.53  (0.75, 3.13) | 4.7 (1.9, 11.1) | 5.33  (0.77, 37.14) | 22.0 (15.9, 29.7) | 1.38  (0.79, 2.42) | 14.9 (9.9, 21.8) | 1.15  (0.52, 2.54) | 29.5 (22.3, 37.9) | 1.36  (0.83, 2.22) |
|  | 200-399% | 5.3 (2.6, 10.6) | 1.39  (0.49, 3.90) | 9.5 (6.3, 14.2) | 1.06  (0.57, 1.97) | 2.7 (1.0, 7.4) | 3.11  (0.54, 17.96) | 11.7 (7.9, 16.9) | 0.92  (0.54, 1.59) | 11.2 (7.5, 16.3) | 1.10  (0.58, 2.09) | 18.3 (13.8, 23.9) | 1.01  (0.66, 1.55) |
|  | ≥400% | 3.4 (1.8, 6.6) | ref | 7.6 (5.0, 11.5) | ref | 1.1 (0.2, 5.4) | ref | 10.8 (7.7, 15.0) | ref | 8.2 (5.2, 12.7) | ref | 15.8 (11.8, 20.9) | ref |
| Primary Caregiver Marital Status^¶^ | Married or living with a partner | 4.9 (3.3, 7.1) | ref | 10.8 (8.5, 13.5) | ref | 2.7 (1.5, 4.9) | ref | 13.4 (10.9, 16.4) | ref | 11.0 (8.7, 13.9) | ref | 19.7 (16.8, 22.9) | ref |
|  | Never married | 3.3 (1.3, 8.2) | 0.46  (0.16, 1.37) | 14.8 (6.6, 30.1) | 0.88  (0.35, 2.26) | 1.2 (0.2, 6.4) | 0.23  (0.03, 1.61) | 21.8 (12.1, 36.2) | 1.16  (0.58, 2.30) | 7.6 (3.0, 17.9) | 0.40  (0.16, 1.03) | 26.1 (15.2, 41.0) | 0.95  (0.52, 1.73) |
|  | Divorced, separated, or widowed | 8.9 (4.4, 17.1) | 1.59  (0.76, 3.34) | 13.0 (7.5, 21.4) | 1.00  (0.58, 1.75) | 3.1 (1.2, 7.9) | 0.97  (0.35, 2.66) | 18.1 (11.8, 26.9) | 1.17  (0.74, 1.86) | 8.2 (4.7, 13.9) | 0.64  (0.37, 1.13) | 22.0 (14.9, 31.3) | 0.98  (0.66, 1.47) |
| Highest Level of Caregiver Education^#^ | High school or less than high school | 7.5 (3.8, 14.1) | 1.97  (0.80, 4.87) | 17.6 (11.7, 25.6) | 2.37  (1.36, 4.11) | 3.7 (1.5, 8.8) | 1.22  (0.35, 4.24) | 23.6 (16.7, 32.1) | 2.29  (1.46, 3.58) | 12.7 (8.2, 19.1) | 1.63  (0.93, 2.85) | 27.9 (20.4, 36.8) | 1.85  (1.28, 2.68) |
|  | Some college or Associate Degree | 7.1 (4.2, 11.6) | 1.80  (0.85, 3.81) | 13.9 (10.1, 18.9) | 1.80  (1.11, 2.92) | 1.7 (0.8, 3.9) | 0.52  (0.16, 1.67) | 16.3 (12.2, 21.4) | 1.55  (1.03, 2.33) | 13.8 (9.7, 19.3) | 1.70  (1.02, 2.82) | 25.4 (20.3, 31.3) | 1.63  (1.20, 2.22) |
|  | College degree or higher | 3.7 (2.3, 6.1) | ref | 7.4 (5.3, 10.3) | ref | 2.5 (1.2, 5.4) | ref | 10.1 (7.7, 13.1) | ref | 7.5 (5.3, 10.4) | ref | 14.9 (12.1, 18.3) | ref |
| Health Insurance Coverage**^\|\|^** | Any private insurance | 4.4 (2.8, 6.9) | ref | 8.0 (6.0, 10.6) | ref | 2.1 (1.0, 4.5) | ref | 10.8 (8.5, 13.7) | ref | 9.0 (6.8, 11.9) | ref | 16.6 (13.9, 19.8) | ref |
|  | Public insurance only | 8.1 (5.0, 12.9) | 1.51  (0.59, 3.87) | 18.1 (13.2, 24.4) | 1.98  (1.11, 3.55) | 4.1 (2.1, 7.9) | 1.37  (0.29, 6.55) | 22.9 (17.4, 29.6) | 1.84  (1.14, 2.97) | 13.9 (9.9, 19.1) | 1.75  (0.91, 3.35) | 29.0 (22.7, 36.1) | 1.60  (1.07, 2.39) |
|  | None | 0.4 (0.1, 1.7) | 0.08  (0.02, 0.39) | 9.1 (3.2, 23.0) | 1.03  (0.34, 3.14) | 0.0 (0.0, 0.3) | 0.01  (0.00, 0.15) | 11.0 (4.4, 24.9) | 0.91  (0.34, 2.43) | 1.7 (0.3, 8.3) | 0.21  (0.04, 1.16) | 13.4 (5.9, 27.4) | 0.76  (0.32, 1.77) |
| ACE Scores^††^ | 0 | 1.9 (1.1, 3.5) | ref | 6.8 (4.8, 9.7) | ref | 1.0 (0.5, 2.2) | ref | 8.8 (6.5, 11.9) | ref | 8.4 (6.1, 11.5) | ref | 14.0 (11.0, 17.5) | ref |
|  | 1 | 6.9 (3.7, 12.4) | 3.08  (1.31, 7.26) | 12.6 (8.4, 18.5) | 1.60  (0.94, 2.71) | 4.2 (1.7, 10.1) | 3.29  (0.99, 10.93) | 12.3 (8.2, 18.0) | 1.21  (0.72, 2.03) | 10.0 (6.3, 15.6) | 1.03  (0.58, 1.86) | 18.4 (13.5, 24.7) | 1.20  (0.81, 1.77) |
|  | 2-3 | 7.6 (4.3, 13.2) | 3.11  (1.20, 8.06) | 13.0 (8.7, 19.1) | 1.33  (0.73, 2.43) | 3.9 (1.5, 9.7) | 2.97  (0.81, 10.87) | 20.6 (14.8, 28.0) | 1.84  (1.12, 3.02) | 16.0 (10.8, 23.2) | 1.60  (0.96, 2.68) | 27.9 (21.0, 36.1) | 1.69  (1.16, 2.45) |
|  | 4 or more | 12.0 (5.5, 24.2) | 5.18  (2.14, 12.51) | 25.4 (15.3, 39.1) | 2.77  (1.52, 5.04) | 2.9 (1.3, 6.0) | 2.60  (0.70, 9.59) | 35.5 (24.6, 48.2) | 3.27  (1.99, 5.36) | 5.0 (2.4, 10.1) | 0.58  (0.26, 1.31) | 38.5 (27.4, 50.9) | 2.45  (1.62, 3.71) |

ACE: adverse childhood experiences; aPR: adjusted prevalence ratio; autism: autism spectrum disorder; CI: Confidence Interval; FPL: Federal Poverty Level; NH: non-Hispanic

^†^Any current autism, developmental delay, intellectual disability, learning disability and/or speech or other language disorder

^‡^Confidence interval calculated using Taylor Series

^§^No confounders identified for adjustment

^¶^Adjusted for race and ethnicity and highest level of caregiver education

^#^Adjusted for race and ethnicity

**^||^**Adjusted for family income

^††^Adjusted for race and ethnicity, age, family income, primary caregiver marital status, highest level of caregiver education, health insurance coverage

**Supplemental Table S5. Prevalence of Mental, Behavioral, or Developmental Disorders Among Children 6-17 Years Old with and Without Heart Conditions, National Survey of Children’s Health, United States, 2016-2019 (Sensitivity Analysis Excluding the Years 2020 and 2021 Affected by the COVID-19 Pandemic)**

|  | **Heart Condition** | | | **No Heart Condition** | |  |
| --- | --- | --- | --- | --- | --- | --- |
| **Characteristic** | | **N** | **Weighted Percent (95% CI**^†^**)** | **N** | **Weighted Percent, (95% CI**^†^**)** | **aPR (Heart Condition v. No Heart Condition)**^‡^  **(95% CI)** |
| ≥1 Mental, Behavioral, or Developmental Disorder^§^ | 887 | | 40.5 (36.3,44.9) | 20,234 | 22.5 (21.9,23.1) | 1.76  (1.58, 1.96) |
| Depression | 220 | | 7.9 (6.1,10.2) | 4,065 | 4.0 (3.8,4.3) | 1.85  (1.41, 2.42) |
| Anxiety Problems | 478 | | 20.9 (17.8,24.5) | 9,232 | 9.1 (8.8,9.5) | 2.19  (1.84, 2.60) |
| ADHD | 415 | | 18.8 (15.7,22.4) | 9,436 | 10.1 (9.7,10.5) | 1.81  (1.52, 2.15) |
| Behavioral or Conduct Problems | 347 | | 15.7 (13.0,18.8) | 6,206 | 7.4 (7.1,7.8) | 2.03  (1.68, 2.45) |
| Tourette syndrome | 14 | | 0.5 (0.3,1.0) | 228 | 0.2 (0.2,0.3) | 2.18  (1.11, 4.25) |
| ≥1 Mental, Emotional, or Behavioral Disorder^¶^ | 728 | | 31.8 (28.0,35.9) | 16,958 | 18.1 (17.5,18.6) | 1.71  (1.50, 1.93) |
| Autism | 132 | | 7.3 (5.2,10.2) | 2,370 | 3.0 (2.7,3.4) | 2.39  (1.69, 3.39) |
| Developmental Delay | 298 | | 15.4 (12.6,18.6) | 3,824 | 4.7 (4.4,5.0) | 3.20  (2.62, 3.92) |
| Intellectual Disability | 96 | | 5.6 (3.9,8.0) | 770 | 1.0 (0.9,1.2) | 5.50  (3.75, 8.06) |
| Learning Disability | 383 | | 19.1 (15.9,22.7) | 6,078 | 7.3 (6.9,7.7) | 2.57  (2.14, 3.08) |
| Speech or Other Language Disorder | 242 | | 12.8 (10.3,15.7) | 3,314 | 4.5 (4.1,4.8) | 2.81  (2.25, 3.50) |
| ≥1 Developmental Disorder^#^ | 508 | | 25.6 (22.0,29.5) | 8,967 | 11.0 (10.5,11.5) | 2.28  (1.97, 2.64) |

aPR: adjusted prevalence ratio; autism: autism spectrum disorder; CI: confidence interval

^†^Confidence interval calculated using Taylor Series

^‡^Adjusted for child’s sex, race and ethnicity, income, and the highest level of education in the household

^§^Any current depression, anxiety problems, ADHD, behavioral or conduct problems, Tourette syndrome, autism, developmental delay, intellectual disability, learning disability and/or speech or other language disorder

^¶^Any current depression, anxiety problems, ADHD, behavioral or conduct problems, and/or Tourette syndrome

^#^Any current autism, developmental delay, intellectual disability, learning disability and/or speech or other language disorder
